# Non-destructive estimation of individual buckwheat plant biomass using high-throughput phenotyping-derived canopy traits

**DOI:** 10.3389/fpls.2026.1953140

**Published:** 2026-10-08

**Authors:** Stephen Njehia Njane, Takashi Hara, Kumpei Ota, Hiroshi Nakagawa

**Affiliations:** 1 Research Centre for Agricultural Information Technology, National Agriculture and Food Research Organization (NARO), Tsukuba, Japan; 2 Hokkaido Agricultural Research Center, National Agriculture and Food Research Organization (NARO), Kasaigun, Japan

**Keywords:** biomass estimation, buckwheat, HTP-derived crop traits, multi-camera system, radiation use efficiency

## Abstract

In this research, we aim to develop a technique for estimating biomass of individual buckwheat from high-throughput phenotyping derived crop traits (HTP-derived crop traits). A tractor-mounted multi-camera phenotyping system was utilised to take images which were processed to generate crop height, volume index and coverage. The accuracy of the imaging system was first validated by comparing image-derived plant height with manual measurements, showing strong agreement with high linear coefficients of determination (R^2^) of 0.95 and 0.93 for “18sd-1” and “Kitamitsuki”, respectively. A biomass estimation model was then developed by relating biomass accumulation to estimated HTP-derived crop traits and compared to that of Monteith-based RUE framework. Biomass estimation was evaluated using one-stage, two-stage, and three-stage models, where the two-stage model separated pre- and post-flowering periods and the three-stage model further divided the pre-flowering period into early and mid-growth stages. The three-stage model improved performance, indicating that developmental stage should be considered when modelling biomass accumulation in buckwheat. Among the evaluated HTP-derived traits, volume index showed the strongest association with measured biomass and showed a similar accuracy as that of the conventional coverage-based RUE framework. These results demonstrate that HTP-derived structural traits, particularly volume index, provide a practical and biologically interpretable approach for repeated, non-destructive estimation of individual-plant biomass in buckwheat.

## 1 Introduction

Buckwheat (*Fagopyrum esculentum* Moench) is an important traditional crop in Japan and remains in steady demand despite domestic production being lower than national consumption (Ohsawa, 2020). It is believed to have been introduced to Japan via the Korean peninsula from China, and it became widely valued because of its rapid growth and adaptability to relatively infertile land (Mikami et al., 2018). Its agronomic value lies in its short growth duration, adaptability to relatively infertile soils, and suitability for diversified cropping systems. In addition, buckwheat is recognized for its nutritional and functional properties, including its content of vitamins, polyphenols, flavonoids, and essential amino acids (Jung et al., 2015; Suzuki et al., 2020). Owing to its starch characteristics, it is widely processed into noodles, bread, and confectionery products (Hung et al., 2009), and its potential use as a forage resource has also been reported (Amelchanka et al., 2010). In Japan, although buckwheat production declined during the expansion of high-yielding rice cultivation, it has regained importance in recent decades, partly due to policy measures promoting conversion of paddy land to alternative crops (Mikami et al., 2018; Ohsawa, 2020).

Hokkaido accounts for a major share of buckwheat production in Japan, and “Kitawasesoba” has long been the dominant cultivar in the region (Honda and Abe, 1992). However, stable buckwheat production remains difficult because the crop is highly sensitive to environmental disturbances, particularly lodging, shattering, and yield instability associated with heavy rainfall and typhoon events (Suzuki et al., 2023). As a result, the average buckwheat yield in Hokkaido over the past 10 years has been only about 68 kg/10 a, which is significantly lower than major crops. For these reasons, buckwheat is classified as a neglected and underutilized species. Breeding for stable, high-yielding cultivars is therefore paramount. However, this is complicated by the reproductive biology of buckwheat, which is an allogamous species with heteromorphic self-incompatibility controlled by the S-locus. Furthermore, increasing the degree of genetic fixation leads to inbreeding depression. As a result, a single variety of population contains individuals with diverse genotypes, making conventional genetic improvement more complex than in self-pollinated crops. Therefore, efficient selection strategies are required to accelerate progress in buckwheat breeding (Ohsawa, 2020). An important aspect of efficient selection strategies is to select individual plants before flowering to prevent random cross-pollination. Although pre-flowering genomic selection has shown promise for yield improvement in buckwheat, precise phenotyping remains indispensable for identifying superior growth- and yield-related traits at the individual plant level (Yabe et al., 2018).

Hitherto, evaluation of buckwheat growth traits has relied primarily on manual and often destructive measurements. Traits such as plant height, stem thickness, number of nodes, leaf characteristics, and biomass components are typically assessed during or after harvest, and biomass is commonly estimated from destructive sampling of leaves and branches (Jung et al., 2015). Furthermore, these trait values are presented as average values of a small number of individuals. Among these traits, biomass is of particular importance because of its close association with yield and the difficulty of assessing it non-destructively before flowering. While such approaches are informative in conventional buckwheat breeding, which involves post-harvest phenotypic selection, precise phenotypic analysis of large numbers of individuals is labour-intensive, time-consuming, and unsuitable for repeated assessment of growth dynamics throughout the crop cycle. Therefore, non-destructive phenotyping approaches are needed to enable repeated and precise characterization of plant growth over time, phenotypic evaluation of numerous individuals, and phenotypic selection before flowering.

Recent advances in remote sensing have expanded the possibilities for crop monitoring in buckwheat and other species. For example, UAV-based RGB imagery has been used to predict buckwheat maturity through extraction of vegetation indices and integration with regression models (Wu et al., 2022). Similarly, UAV-derived RGB data have been applied to estimate chlorophyll values in barley, although the strength of association depended on image acquisition height (Liu et al., 2021). Although these studies demonstrate the value of image-based sensing for crop assessment, most remote sensing approaches have been developed for field-scale monitoring and are not optimized for precise phenotyping of individual plants. This limitation is particularly relevant in buckwheat, where dense canopies and lodging-prone architecture complicate accurate trait extraction. For breeding purposes, plant-level phenotyping is especially important because selection is ultimately based on differences among individual genotypes or plants rather than on field-level estimates.

In parallel with sensing-based approaches, crop growth models have been widely used to simulate crop development, biomass accumulation, and yield formation by representing interactions among genotype, environment, and management. Among these, APSIM has been applied to evaluate conservation agriculture effects on soil properties, assess wheat adaptation in semi-arid environments, simulate crop growth, and improve yield prediction when combined with machine learning approaches (Brown et al., 2018; Briak and Kebede, 2021; Li et al., 2024; Yadav et al., 2025). Likewise, WOFOST has been used to estimate aboveground biomass and soil water content, assess the effects of low-temperature stress, and analyse soil moisture–yield relationships in cereal systems (Bergjord Olsen et al., 2018; Dewenam et al., 2021; Chen et al., 2024; Zhang et al., 2025). While such models are powerful for describing crop growth dynamics at the canopy or field scale, they generally do not capture detailed plant-level structural traits derived from high-resolution phenotyping. Consequently, there remains a gap between image-based phenotyping and simple mechanistic modelling frameworks for biomass estimation.

Radiation use efficiency (RUE)-based modelling provides a useful intermediate framework for linking plant structure and biomass accumulation. In the classical Monteith formulation, biomass production is related to the amount of intercepted radiation and the efficiency with which that radiation is converted into dry matter (Osman, 1971; Monteith, 1972; Landsberg and Hingston, 1996). Such an approach offers a relatively simple but biologically interpretable means of biomass prediction, especially when suitable proxies for canopy development and light interception are available (Vieira et al., 2009). In this context, image-derived phenotypic traits such as canopy coverage and volume-related indices may provide an effective basis for estimating variation in light interception and growth among individual buckwheat plants.

Although high-throughput phenotyping has shown potential for non-destructive crop monitoring, its application to individual-plant biomass estimation in buckwheat remains limited (Filho et al., 2020). showed that the fresh weight of buckwheat cultivars could be estimated using an optimal plot size of 8 m^2^, enabling treatment differences among plots to be detected. However, this approach did not allow individual-plant differences within buckwheat populations to be evaluated (Antala et al., 2025). examined the response of individual buckwheat plants to water stress using a phenotyping platform, but their study focused mainly on physiological traits such as plant height and vegetation indices as proxies for biomass, without directly validating their relationship with biomass.

In this study, we aimed to develop a practical framework for estimating individual-plant biomass in buckwheat using HTP-derived crop traits. A tractor-mounted multi-camera system was used to acquire RGB images, which were processed to extract plant height, canopy coverage, and volume index at the individual-plant level. The accuracy of the system was first evaluated by comparing image-derived plant height with manual measurements. Biomass estimation models were then developed using the extracted HTP-derived traits and compared with a conventional Monteith-based radiation use efficiency framework. Finally, stage-wise models were evaluated to determine whether incorporating developmental stage, particularly flowering stage, improved biomass estimation.

## 2 Materials and methods

### 2.1 Plant materials and greenhouse experiment

The experiment was conducted at the Hokkaido Agricultural Research Center, National Agriculture and Food Research Organization (NARO), Japan. Buckwheat plants were grown in cultivation tanks (PSK-6000A, Yazaki Kako Corp., Shizuoka, Japan) under greenhouse conditions. Each cultivation tank was filled with culture soil (Peat pot V, 300 mg/L Nitrogen, 1,200 mg/L Phosphoric acid, 300 mg/L Potassium, pH 6.0-7.0, Hokkaido Peatmoss Inc., Saitama, Japan). Two genotypes with contrasting plant shape were used to evaluate the versatility of the tractor-mounted multi-camera phenotyping system: the cultivar “Kitamitsuki” and the semidwarf line “18sd-1”. Sowing was carried out on 30th May 2025. Irrigation was applied using narrow-spray irrigation tubes (EVAFLOW, MKV Advance Co., Ltd., Japan) whenever the soil surface had dried.

For each genotype, the relationships between HTP-derived crop traits and manually measured crop traits were analysed. Image acquisition and manually measured were conducted at eight time points: 17, 19, 21, 24, 26, 28, 31, and 33 days after planting (DAP). Separate groups of plants were established in advance for destructive sampling at each measurement period. To minimize overlap among neighbouring plants as canopy size increased with development, plants designated for sampling at 17, 19, and 21 DAP were grown at 20-cm intervals, those designated for 24, 26, and 28 DAP were grown at 40-cm intervals, and those designated for 31 and 33 DAP were grown at 60-cm intervals. Plant spacing was fixed from establishment and was not altered during growth. At each DAP, manual measurements were obtained by recording the main stem length, weights of stems and leaves collected from the base of plants. The collected plant material was dried for 10 days in a drying room maintained at approximately 35 °C and 20% relative humidity before weighing. Although constant weight was not formally confirmed through repeated weighing, the samples were dried to a stable dry state under these conditions. Thus, the reported biomass represents dry weight standardized across samples using the same 10-day drying procedure. Tractor-mounted multi-camera phenotyping system.

A tractor-mounted imaging platform was developed for non-destructive phenotyping of buckwheat plants in the greenhouse. The platform consisted of an MR1000AH robot tractor (Kubota Corp., Japan) fitted with a horizontal multi-camera arm carrying six RGB cameras (**Figures 1a, b**). The tractor was guided automatically using a real-time kinematic global navigation satellite system (RTK-GNSS), whereby an AB line was established along the length of the greenhouse to enable autonomous movement during image acquisition.

**Figure 1:**
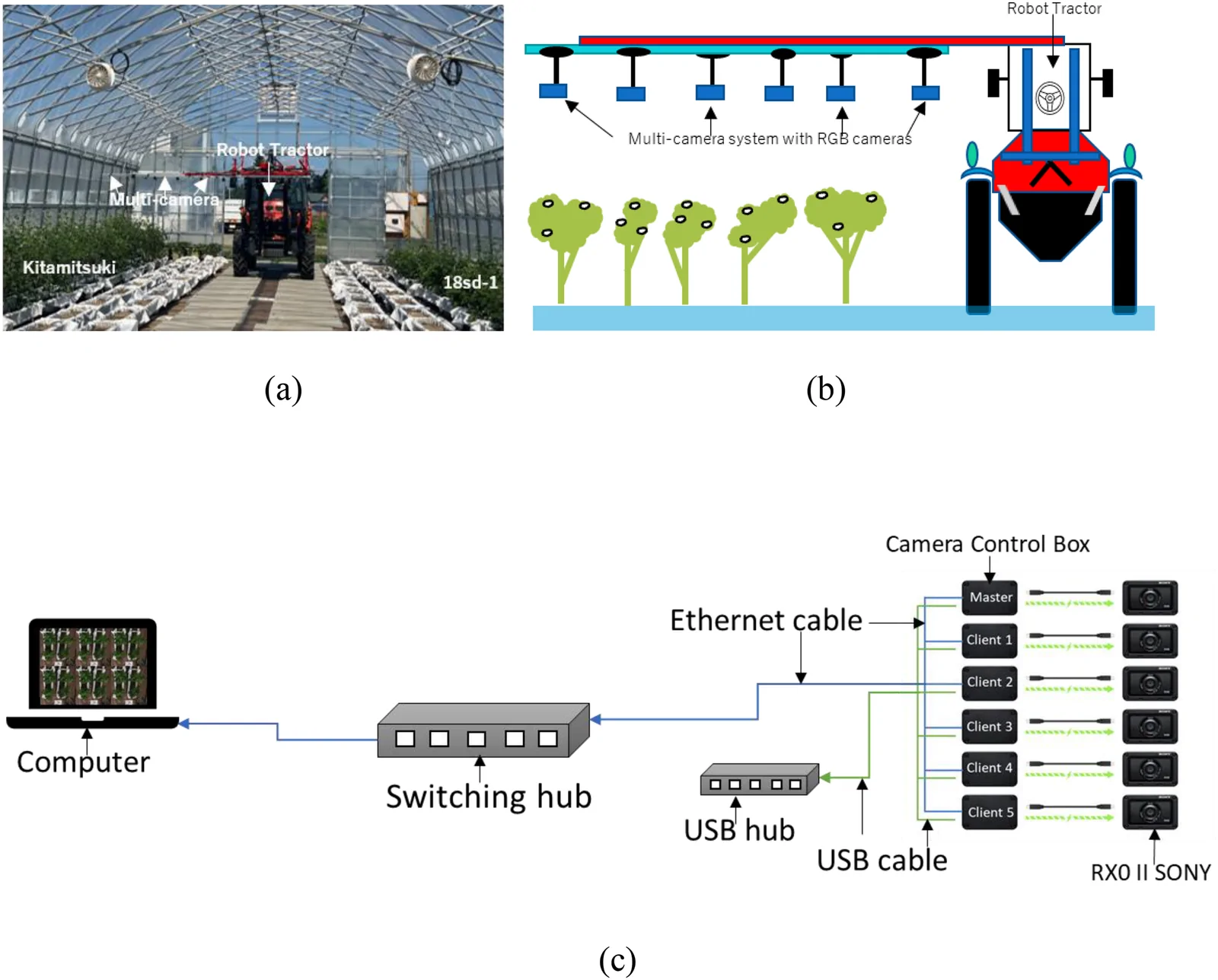
Experimental set up in the greenhouse with **(a)** the multi-camera fitted robot tractor moving along the rows of crops and **(b)** the set-up of the RGB cameras and **(c)** multi-camera system with camera control boxes and PC for within-cabin shutter control.

At the start of image acquisition, the tractor entered the greenhouse and stopped near the entrance, after which the camera arm was extended electrically. Images were acquired while the tractor moved at a speed of 2 km h^−1^. Imaging was first conducted on the side containing “Kitamitsuki” plants. After this pass, the arm was retracted, the tractor exited the greenhouse, made a U-turn, and re-entered to acquire images of the opposite side containing “18sd-1” plants. Since the camera arm was mounted only on the right side of the tractor, this procedure was necessary to take images from both sides of the greenhouse.

The multi-camera unit consisted of six RX0 II cameras (DSC-RX0M2, Sony Corp., Japan), each equipped with a 15.3-megapixel sensor, a focal length of 7.9 mm, and a field of view of 84°. The cameras were mounted in nadir orientation at 45-cm intervals along an adjustable horizontal arm positioned at the front of the tractor. Image acquisition was conducted using a shutter speed of 1/1000 s with automatic ISO. To ensure sufficient image overlap for subsequent three-dimensional reconstruction, the camera arm was positioned 2.25 m above the ground surface, following a previously reported configuration by Njane et al. (2024), and images were captured at 1-s intervals. Images were acquired thrice per week from sowing until maturity.

To enable remote and synchronized control of the cameras, each camera was connected to a camera control box (CCB-WD1, Sony Corp., Japan) as shown in **Figure 1c**. One control box served as the master unit, while the remaining five functioned as client units receiving settings from the master. All control boxes were connected via Ethernet cables to a switching hub, which in turn was connected to a computer located in the tractor cabin. Through IP-based communication, the cameras were operated from inside the tractor cabin using a browser-based user interface, enabling remote shutter control and synchronized adjustment of camera settings.

### 2.2 Image processing and map generation

The RGB images acquired by the multi-camera system were processed in Agisoft Metashape Professional version 1.2.6 (Agisoft LLC, 2021) to generate spatial products for crop trait extraction. Image reconstruction was performed using a structure-from-motion and multi-view stereo (SfM/MVS) workflow. This procedure included image alignment, dense point cloud generation, and subsequent construction of digital elevation models (DEMs) and orthomosaic images.

For each genotype, images were processed separately. Images acquired from the rows containing “Kitamitsuki” and “18sd-1” plants were first imported into Metashape and aligned. Pre-measured ground control points (GCPs), obtained from markers placed at alternating positions inside the greenhouse, were imported as CSV files to improve georeferencing accuracy. A batch processing workflow was then applied to generate a dense point cloud, mesh, DEM, and RGB orthomosaic. The resulting DEMs and RGB orthomosaics were exported for downstream phenotypic analysis.

Following three-dimensional reconstruction, the generated point clouds, DEMs, and orthomosaic images were visually inspected for reconstruction quality before phenotypic trait extraction. Unreliable reconstruction was identified from features such as incomplete reconstruction of the plant canopy, obvious gaps or distortions in the reconstructed plant structure, and inconsistencies between the reconstructed surface and the corresponding RGB imagery. Particular attention was given to volume index because this trait depends directly on the reconstructed three-dimensional canopy surface. At 24 DAP, substantial plant movement during image acquisition resulted in poor reconstruction quality across the dataset, and the data from this date were therefore excluded from further analysis. No other complete measurement-date dataset was excluded.

### 2.3 Crop trait extraction

Until recently, extraction of HTP-derived crop traits from generated spatial maps was limited because it required advanced programming skills and complex computer-environment setup, which can be a bottleneck, especially for crop breeders. To overcome this limitation, HTP-derived crop traits were extracted from the generated DEMs and RGB orthomosaic using the Windows-based PREPs (Precision Plots Analyzer) software, which we developed recently for high-throughput extraction of plant phenotypic traits such as crop height, volume index, and coverage from spatial image products (Itoh et al., 2024).

Because individual plants were grown at fixed intervals, microplots corresponding to each plant were delineated prior to trait extraction. Shapefiles representing individual plant regions were generated within PREPs based on the orthomosaic images, enabling segmentation of the spatial products into plant-level units for analysis.

Plant height was estimated from the DEM. In general, crop height is derived from the elevation difference between the top of the canopy and a reference ground surface. In the present experiment, however, the plants were grown in raised rectangular cultivation tanks, and the soil surface within the tanks varied slightly in elevation and inclination as shown in **Figure 2a**. Consequently, using the greenhouse floor as the reference surface would not have provided accurate plant height estimates. To address this, a base plane was defined for each cultivation tank using a rectangular region at the top surface of the pot before plant emergence. This base plane was then used as the reference surface for calculating plant height throughout the growth period as shown in **Figure 2b**.

**Figure 2:**
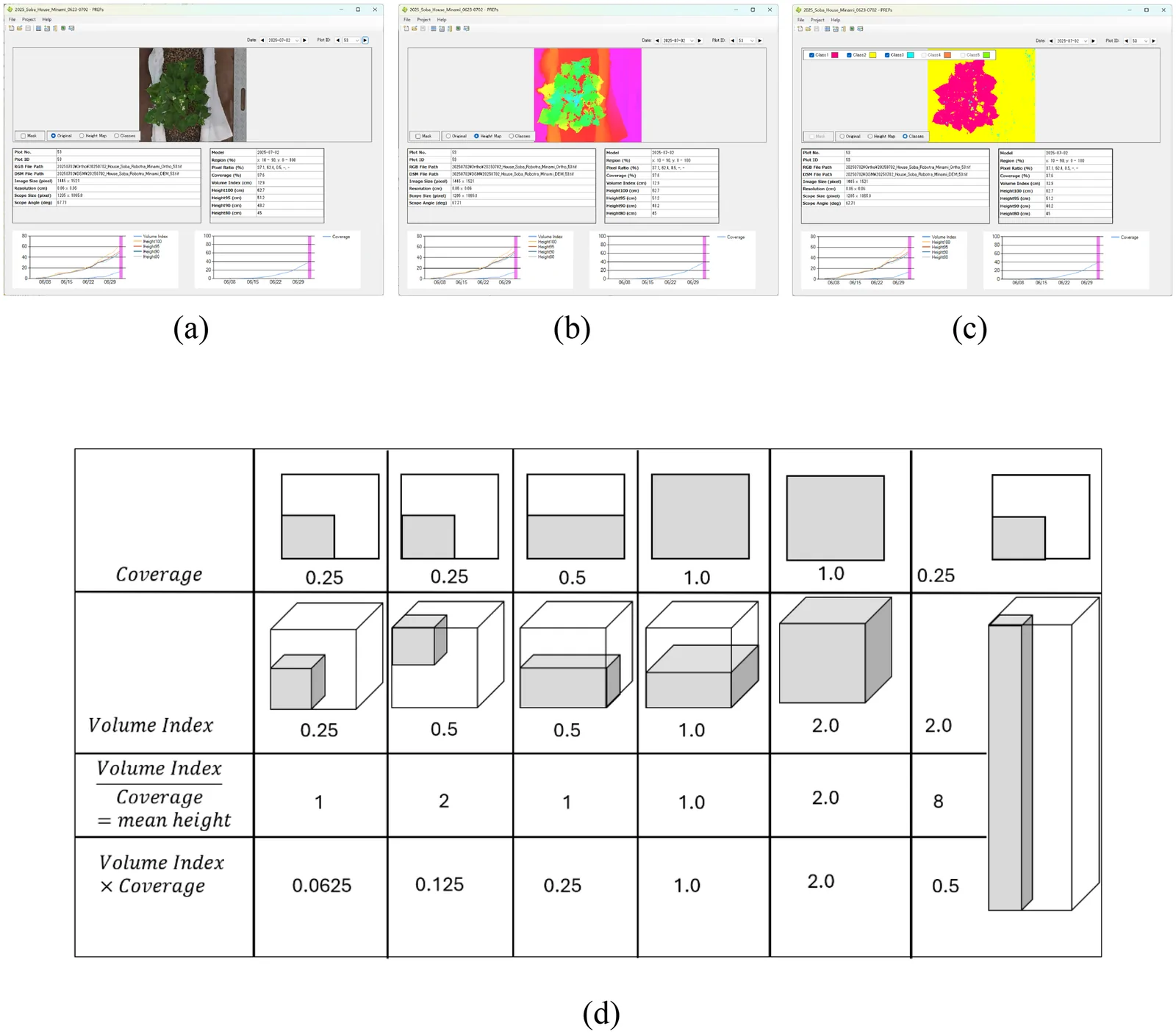
Processing pipeline of individual plants using PREPs software where **(a)** shows the actual crop images **(b)** crop height estimated from the surface of the pot as the base plane and **(c)** coverage estimated by developing a segmentation model that separates the crops from the soil **(d)** is an illustration showing how the HTP-derived crop traits are estimated from the pixels of each of the cell grid of the generated maps.

Canopy coverage was estimated by segmenting vegetation pixels from soil pixels in the orthomosaic images. A segmentation model was generated by assigning crop pixels to one class and soil pixels to another. The trained model was then applied to the orthomosaic to separate vegetation from background soil, and canopy coverage was calculated as the proportion of vegetation pixels within each plant-level microplot (**Figure 2c**).

Volume index was calculated from the DEM as a proxy for three-dimensional canopy development. **Figure 2d** illustrates the difference between canopy coverage and volume index at the pixel level. For each plant-level microplot, the vertical extent of crop pixels above the reference plane was integrated across the defined plant area. Thus, volume index incorporates both the horizontal extent of the canopy and its vertical development. However, volume index does not represent the actual physical volume occupied by plant tissues and should therefore be interpreted as an image-derived structural index of overall canopy development rather than as a direct measurement of true canopy volume.

### 2.4 Radiation use efficiency estimation

Solar radiation was measured using an SR20-D2 (Hukx Ltd, Japan) pyranometer installed at the Hokkaido Agricultural Research Center, Memuro. The sensor measures solar radiation over a 180°field of view and outputs irradiance in W m^−2^. These values were converted into total incoming solar radiation. Irradiance was measured every second in W m^−2^ and averaged over 60-min intervals. The recorded irradiance was integrated over time as shown in Equation 1 and converted to daily incoming solar radiation as:

(1) $$R_{d}=\frac{\sum_{j=1}^{n}I_{j}\text{Δ}t}{10^{6}},$$

where *I_j_* is irradiance at recording interval *j* in W m^−2^, Δ*t* is the recording interval in seconds, and *R_d_* is daily incoming solar radiation in MJ m^−2^ day^−1^. Incident radiation inside the greenhouse was estimated as 0.9*R_d_*, based on an assumed greenhouse transmissivity of 90% (MKV ADVANCE CO., LTD.). This is because since the pyranometer was installed outside the greenhouse, incident radiation within the greenhouse was estimated by multiplying the measured outdoor radiation by an assumed greenhouse transmissivity of 0.9. Furthermore, because greenhouse transmissivity was not continuously measured during the experiment, this value was treated as a fixed approximation for estimating radiation available within the greenhouse.

Daily intercepted radiation was subsequently estimated as shown in Equation 2:

(2) $$IRAD_{\text{d}}=Coverage_{d}\left(0.9R_{d}\right),$$

Where 0.9*R_d_* represents incident radiation on day *d* and *Coverage_d_* represents canopy coverage on the same day. Cumulative intercepted radiation, *IRAD_cum_*,*_t_* was estimated as the time integral of canopy coverage multiplied by incident radiation as indicated in Equation 3:

(3) $$\;IRAD_{cum,t}=\sum_{d=1}^{t}IRAD_{d}$$

and biomass was expressed according to the Monteith radiation interception framework as shown in Equation 4:

(4) $$Biomass=RUE\times IRAD_{cum,t}$$

Biomass was measured directly as dry weight; therefore, no additional dry-matter conversion factor was applied. Cumulative intercepted radiation was calculated by summing daily values over time andwas expressed in MJ m^−2^. The slope of the regression between aboveground dry biomass and cumulative intercepted radiation was interpreted as apparent RUE and expressed as g dry matter MJ^−1^, assuming that canopy interception depended primarily on ground cover and that vertical canopy structure was not explicitly represented in the basic model.

To assess whether developmental stage affected biomass estimation, the growth period was initially divided according to flowering status. Inspection of the temporal changes in biomass and HTP-derived crop traits further indicated a transition within the pre-flowering period from relatively slow initial growth to more rapid canopy and biomass development. Therefore, the growth period was divided into three stages: early pre-flowering (<20 DAP), mid pre-flowering (21–26 DAP), and post-flowering (27–31 DAP). The early pre-flowering stage represented the period of relatively slow initial vegetative growth, whereas the mid pre-flowering stage corresponded to the period of more rapid canopy development and biomass accumulation before flowering. The post-flowering stage corresponded to the period following the onset of flowering. The same DAP-based stage boundaries were applied to both “18sd-1” and “Kitamitsuki”. As a comparison, the relationship between biomass and volume index, was compared with that of biomass with volume index × coverage and finally that of biomass with Summation volume index × coverage × radiation. Flowering was selected as a biologically meaningful transition because it represents a major developmental stage associated with reproductive growth in buckwheat.

## 3 Results

### 3.1 Accuracy of image-derived crop height

To evaluate the accuracy of the tractor-mounted multi-camera phenotyping system, image-derived crop height was compared with manually measured crop height for both genotypes. In general, “Kitamitsuki” exhibited greater plant height than “18sd-1” throughout the measurement period (**Figure 3**). A strong agreement was observed between manual and image-derived height in both genotypes. For “18sd-1”, the relationship showed a coefficient of determination (R^2^) of 0.95, and a root mean square error (RMSE) of 3.98 cm. For “Kitamitsuki”, the corresponding values were R^2^ = 0.93 and RMSE = 15.59 cm. The slightly lower agreement observed in “Kitamitsuki” may reflect differences in canopy architecture, as this genotype tended to develop taller and more laterally extended branches that were captured by the imaging system, whereas manual measurements primarily represented the height of the main stem. Overall, these results indicate that the multi-camera system provided reliable estimates of crop height for non-destructive monitoring of buckwheat growth under greenhouse conditions.

**Figure 3:**
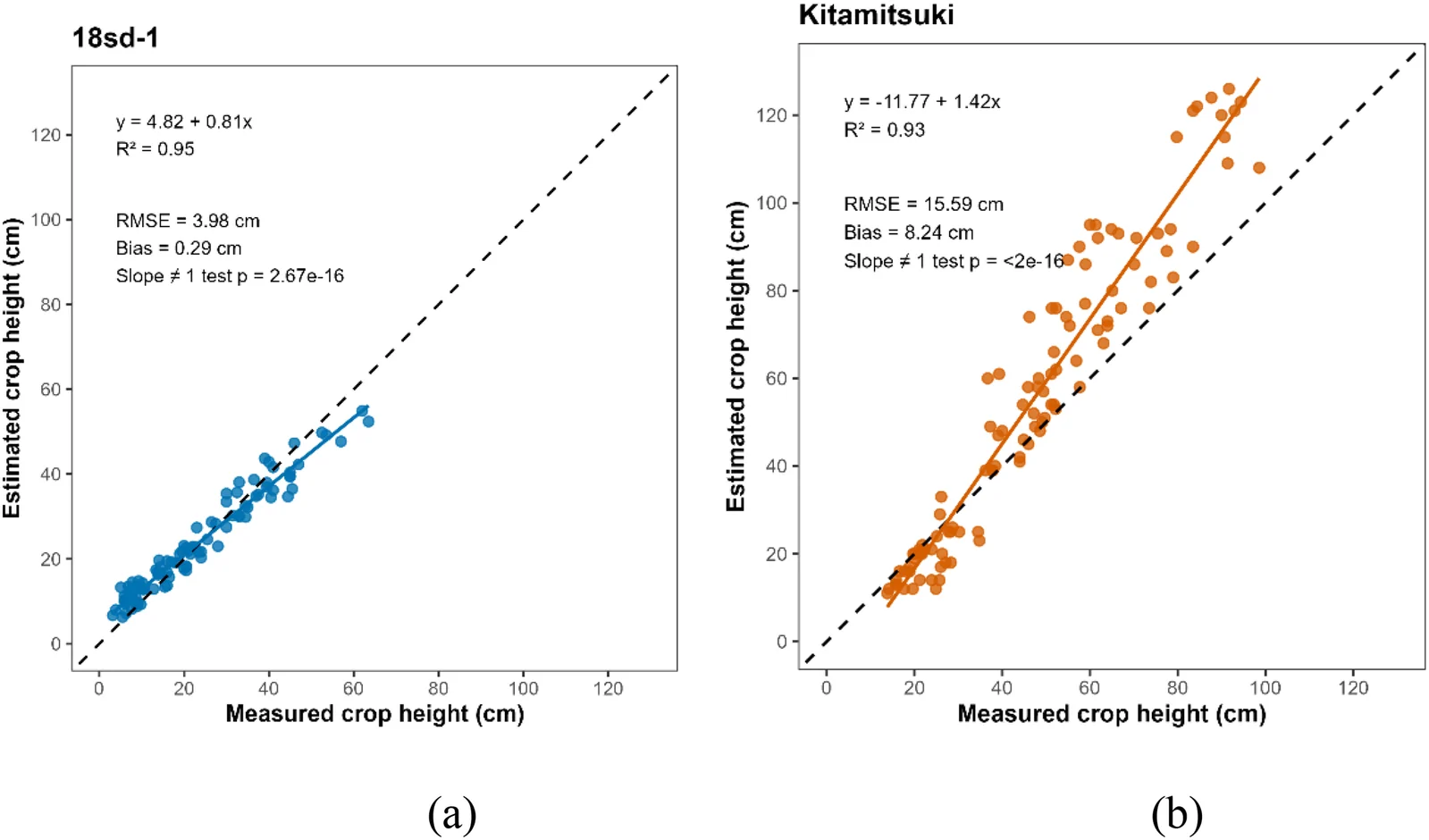
Crop height correlation between manual measurement and crop height estimated by the multi-camera system for both **(a)** ”18sd-1” and **(b)** “Kitamitsuki”.

### 3.2 Temporal changes in biomass and phenotypic traits

Temporal changes in biomass, canopy coverage, and volume index were evaluated for both genotypes (**Figure 4**). Data from 24 DAP were excluded because 3D reconstruction failed. Although 24 DAP occurred within the mid pre-flowering stage (21–26 DAP), measurements at the surrounding time points were available and the overall temporal progression of biomass and HTP-derived crop traits remained evident. Nevertheless, exclusion of this observation reduced the amount of data available for the mid pre-flowering stage and should be considered when interpreting the stage-specific models. Biomass increased progressively over time in both “18sd-1” and “Kitamitsuki”. During the early growth period, up to approximately 22 days after planting, the growth rate was slow, and biomass was similar between the two genotypes. However, from the 24th day onward, the growth rate increased sharply. Moreover, by 24 to 28 days after planting, “Kitamitsuki” exhibited greater biomass than “18sd-1”. This increase corresponded with the more rapid increase in plant height observed in “Kitamitsuki” during later growth stages.

**Figure 4:**
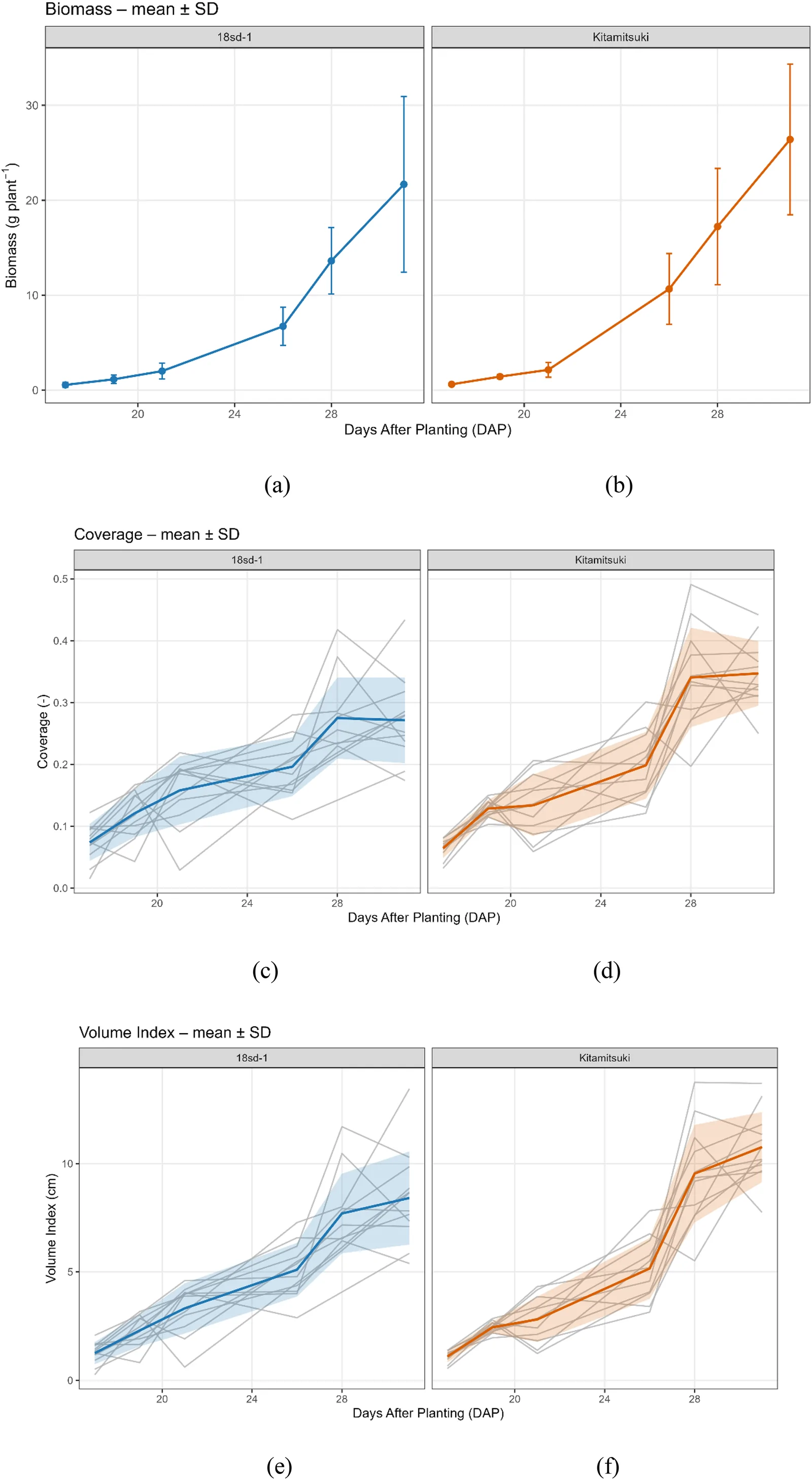
Time-series changes in biomass, canopy coverage, and volume index for “18sd-1” and “Kitamitsuki”. Panels **(a, b)** show biomass, panels **(c, d)** show canopy coverage, and panels **(e, f)** show volume index.

Canopy coverage also increased with time in both genotypes, although the rate of increase became smaller after approximately 28 days after sowing. While both genotypes exhibited similar temporal patterns, canopy coverage was generally higher in “Kitamitsuki” than in “18sd-1”. Changes in volume index showed genotype-specific differences. In “18sd-1”, volume index increased gradually and consistently over time. In contrast, “Kitamitsuki” showed relatively slow initial increase followed by a more rapid increase after approximately 20 days after planting. Although the two genotypes showed similar values during much of the growth period, “Kitamitsuki” tended to exhibit slightly greater volume index at later stages, particularly after 26 days after planting.

### 3.3 Crop trait-biomass

Biomass was compared with estimated HTP-derived crop traits at each DAP to determine the relationship between biomass and each trait. Volume index was highly correlated with biomass, with an R^2^ of 0.95 at 21 DAP, as shown in **Figure 5**. The gradients of the relationships between biomass and volume index, and between biomass and canopy coverage, were then plotted as shown in **Figures 5b, c**. The gradient of the biomass–volume index relationship was higher in “18sd-1” than in “Kitamitsuki”, whereas the biomass–coverage relationship showed similar gradient values in both genotypes.

**Figure 5:**
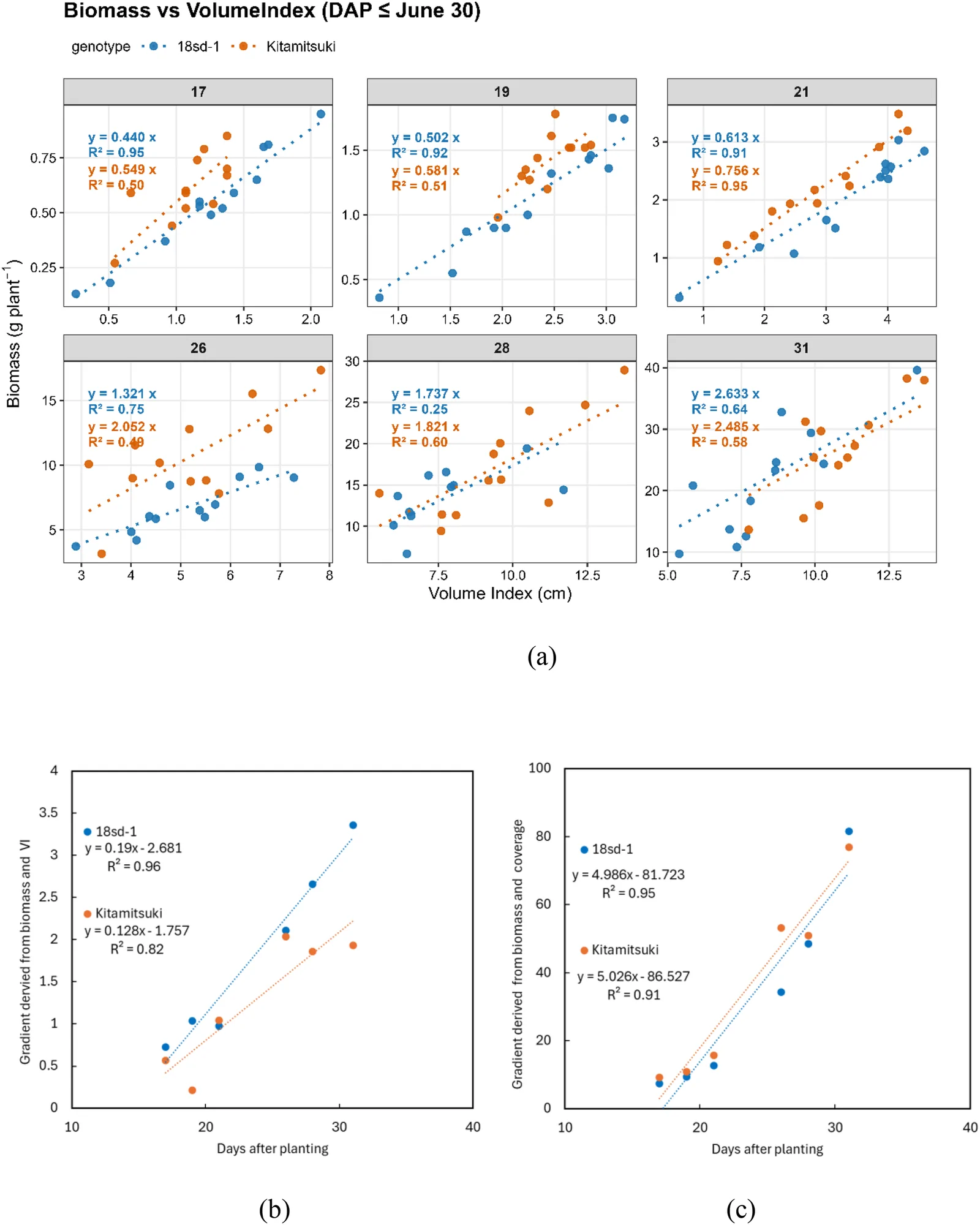
Relationship between biomass and HTP-derived crop traits. Panel **(a)** shows biomass plotted against volume index at different days after planting. Panels **(b, c)** show temporal changes in the gradients of the biomass–volume index and biomass–coverage relationships, respectively.

To evaluate the relationships between HTP-derived crop traits and biomass, regression analyses were performed using biomass as the response variable and combinations of phenotypic traits and radiation-related variables as predictors (**Table 1**). The cumulative product of image-derived traits and radiation was expressed as “Sum,” as shown in **Figure 6**. Incident radiation data measured near the greenhouse facility at the Memuro Research Center were incorporated into the model formulations. In the pooled analysis, volume index showed the strongest relationship with biomass among the HTP-derived crop traits, with the highest coefficient of determination (R^2^ = 0.86) and the lowest RMSE (3.66). Among the models incorporating radiation, Sum VI × C × Radiation showed the best performance, with R^2^ = 0.83 and RMSE = 3.98.

**Table 1:** Comparison of the various model’s combination for both genotypes, Coverage and Volume index are represented as C and VI.

Model	Pooled *R*^2^	Pooled RMSE	18sd-1 *R*^2^	Kitamitsuki *R*^2^	18sd-1 RMSE	Kitamitsuki RMSE
Coverage (C)	0.769	4.645	0.680	0.825	4.919	4.359
Volume Index (VI)	0.857	3.656	0.810	0.886	3.790	3.520
VI × C	0.825	4.038	0.772	0.858	4.153	3.921
Sum C × Radiation	0.805	4.262	0.782	0.817	4.059	4.454
Sum VI × Radiation	0.823	4.070	0.804	0.831	3.848	4.277
Sum VI × C × Radiation	0.830	3.979	0.824	0.831	3.651	4.278

**Figure 6:**
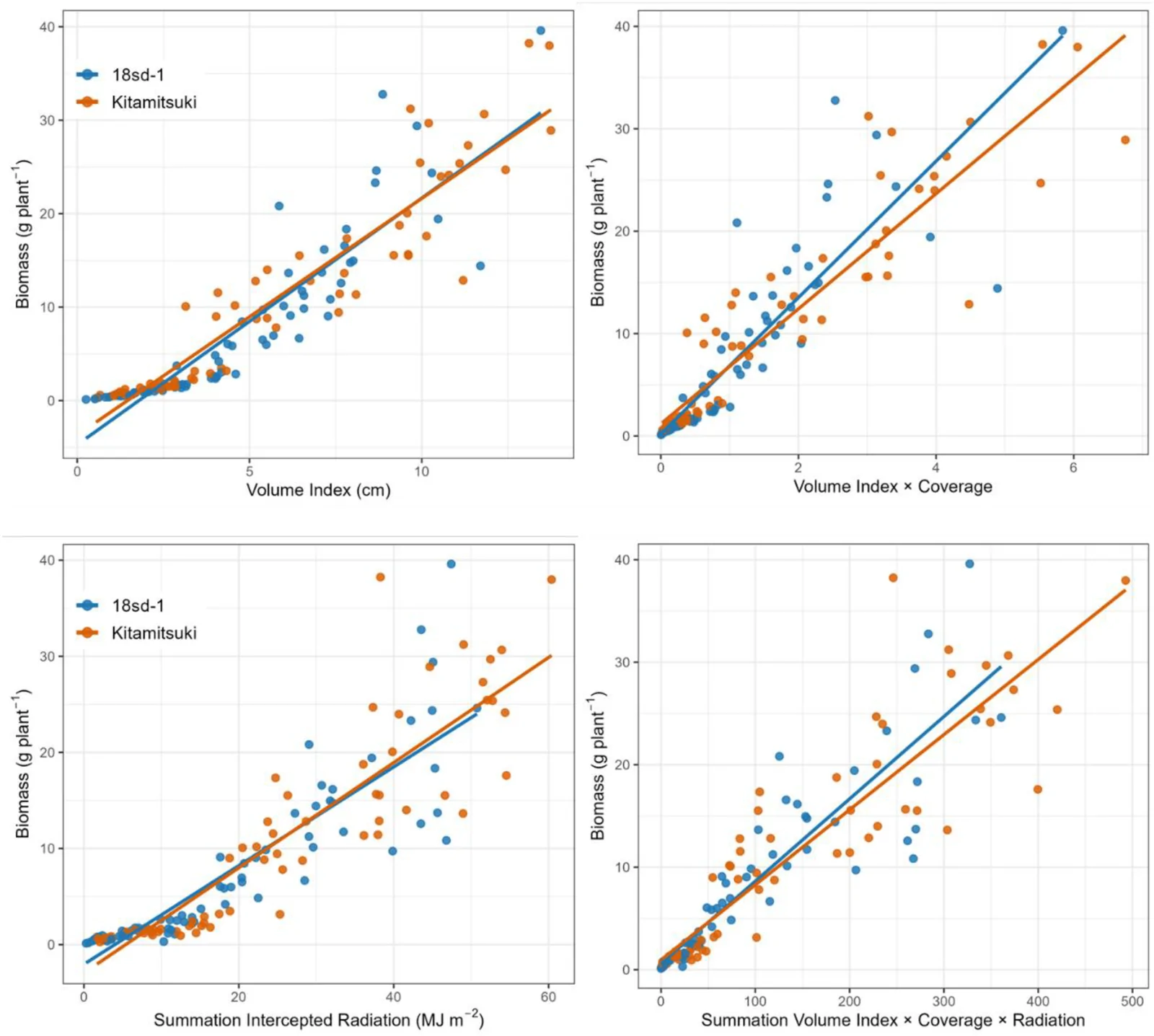
Relationships between biomass and HTP-derived crop traits, and between biomass and combinations of HTP-derived traits with estimated radiation, in “18sd-1” and “Kitamitsuki”.

In both genotypes, most sensing-derived parameters showed moderate to strong relationships with biomass, with generally higher coefficients of determination in “Kitamitsuki” than in “18sd-1”. A comparison of all model combinations is presented in **Table 1**. For “Kitamitsuki”, the strongest relationship with biomass was obtained using Volume Index, which yielded R^2^ = 0.886 and RMSE = 3.52. For “18sd-1”, the best-performing model was Sum VI × C × Radiation, with R^2^ = 0.824 and RMSE = 3.651.

Among the models incorporating only coverage and radiation, Sum C × Radiation produced slopes of 0.513 for “18sd-1” and 0.547 for “Kitamitsuki”, with corresponding R^2^ values of 0.782 and 0.817, respectively. From the pooled result, it was shown that among all the traits and combinations, Volume index had the strongest relationship with biomass with a highest R^2^ = 0.857 and lowest RMSE 3.656.

Because the cumulative product of canopy coverage and radiation was directly related to the radiation use efficiency (RUE) (Arduini et al., 2016) framework adopted in this study, this relationship was examined in greater detail. In both genotypes, cumulative coverage × radiation increased gradually during early growth and more rapidly during later developmental stages. The relationship between biomass and cumulative coverage × radiation formed the basis for subsequent RUE estimation.

### 3.4 Stage-specific RUE estimation

After adjusting incident solar radiation using the assumed greenhouse transmissivity of 0.9, the RUE values estimated using the two-stage model were 0.857 and 1.231 g DM MJ^−1^ for “18sd-1” during the pre- and post-flowering stages, respectively. For “Kitamitsuki”, the corresponding values were 0.953 and 1.027 g DM MJ^−1^.

These results indicate that stage-specific partitioning improved the characterization of biomass accumulation relative to intercepted radiation in both genotypes. Further inspection of the pre-flowering period in the two-stage model showed that this period could be divided into two distinct sub-phases, referred to here as the early and mid-pre-flowering stages, resulting in a three-stage model (**Figure 7**).

**Figure 7:**
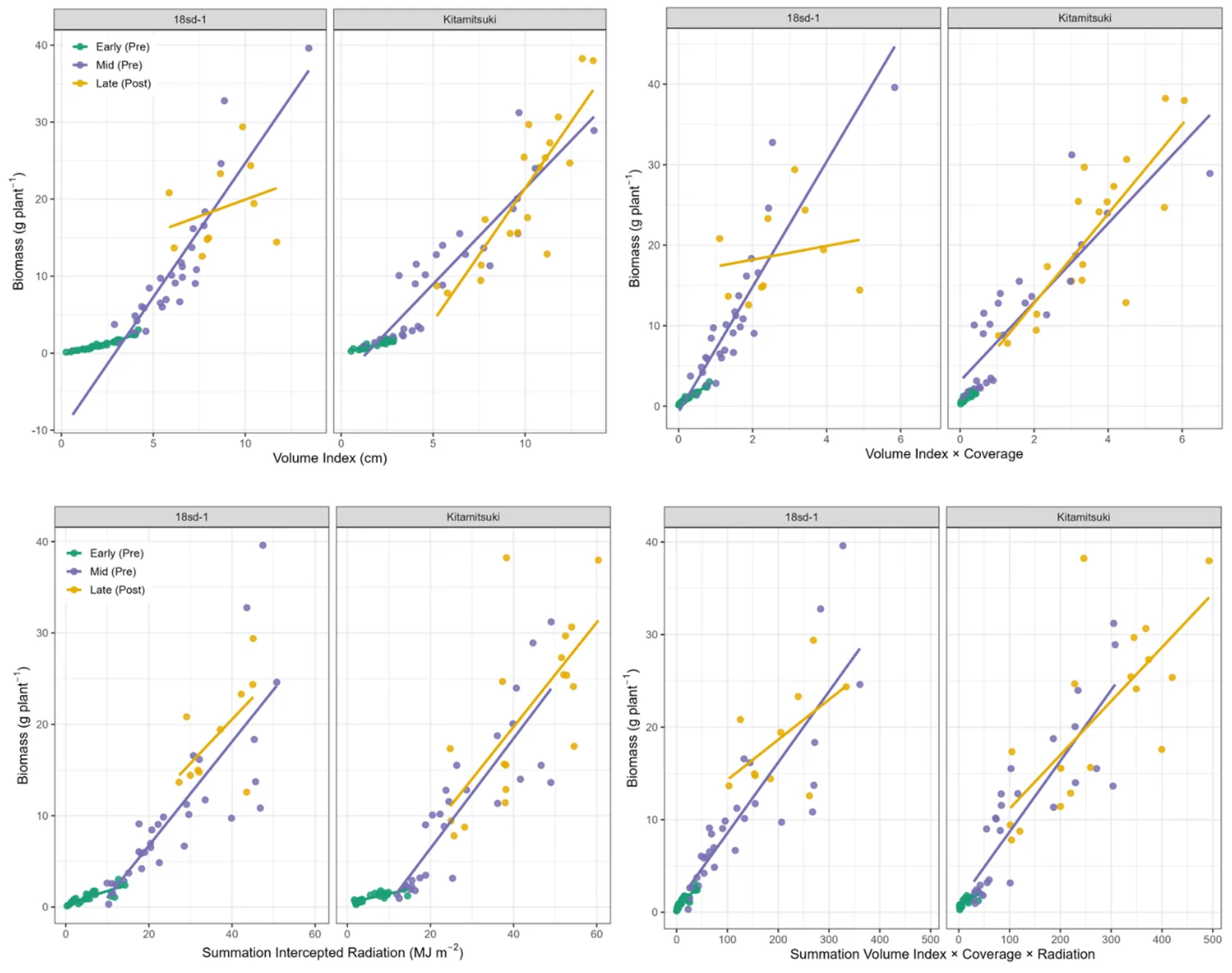
Relationships between biomass and HTP-derived crop traits and their combinations with estimated radiation in both genotypes. The pre-flowering stage was divided into early and mid-growth stages, resulting in a three-stage model.

In both genotypes, RUE was lowest during the early pre-flowering stage and increased substantially during the mid-pre-flowering stage, reaching values comparable to those observed during the post-flowering stage.

Using radiation adjusted for the assumed greenhouse transmissivity of 0.9, the apparent RUE values, excluding the early stage, ranged from approximately 1.0 to 1.23 g DM MJ^−1^.

For “18sd-1”, the converted RUE values were 0.238, 0.994, and 1.232 for the early pre-flowering, mid-pre-flowering, and post-flowering stages, respectively. For “Kitamitsuki”, the corresponding values were 0.279, 1.037, and 1.027.

### 3.5 Biomass estimation performance

Estimated biomass was compared with measured biomass for the three modelling approaches, as shown in **Figure 8a**, where volume index was used as the predictor. Comparison among the models showed that the three-stage model reduced RMSE for “18sd-1” and increased the correlation for “Kitamitsuki”.

**Figure 8:**
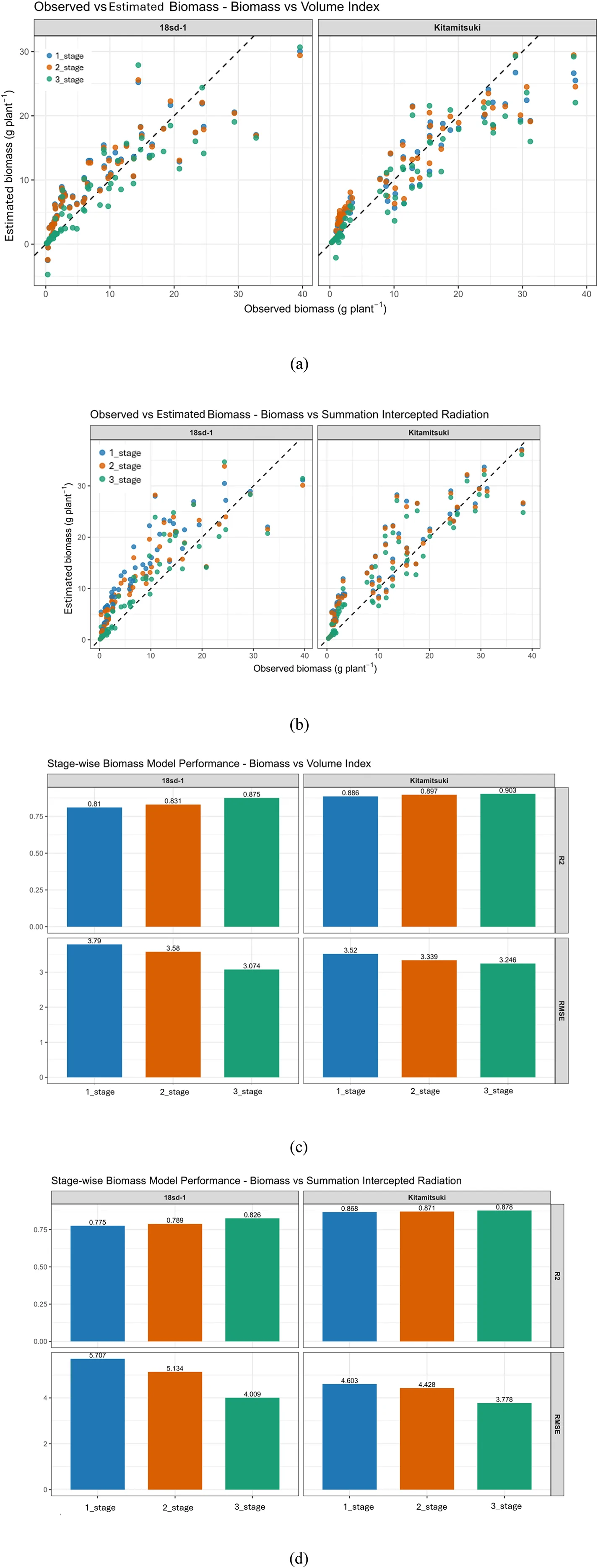
Comparison of observed and predicted biomass using different stage-wise models. Panels **(a, b)** show observed versus predicted biomass for the volume index model and the cumulative intercepted radiation model, respectively. Panels **(c, d)** show the corresponding model performance based on R^2^ and RMSE.

Model performance based on RMSE and R^2^ is summarized in **Figure 8**. For the biomass versus cumulative intercepted radiation model in “18sd-1”, estimation accuracy improved from RMSE = 5.707 and R^2^ = 0.775 in the one-stage model to RMSE = 4.009 and R^2^ = 0.826 in the three-stage model. A similar trend was observed for “Kitamitsuki”, where the one-stage model yielded RMSE = 4.603 and R^2^ = 0.868, while the three-stage model yielded RMSE = 3.778 and R^2^ = 0.878.

However, for the biomass vs volume index model, for “18sd-1”, the best estimation accuracy was improved from RMSE = 3.79 and R^2^ = 0.81 in the 1-stage model to RMSE = 3.074 and R^2^ = 0.875 in the 3-stage model. For “Kitamitsuki”,1-stage model yielded RMSE = 3.52 and R^2^ = 0.886, and 3 stage model yielded RMSE = 3.246 and R^2^ = 0.903. Overall, it was observed that volume index produced the highest coefficient of determination, R^2^ and lowest RMSE in both “18sd-1” and “Kitamitsuki” in the development stage model, thus showing a high potential for utilization in estimation of biomass around the flowering stage.

## 4 Discussion

The present study demonstrated that a tractor-mounted multi-camera phenotyping system can be used to estimate the growth of individual buckwheat plants with high accuracy under greenhouse conditions. In particular, the strong agreement between manually measured and image-derived plant height indicates that the developed system is suitable for non-destructive monitoring of buckwheat growth. Previous work has shown that stereo-vision approaches can be used to estimate buckwheat plant height under field conditions; however, their performance may be limited by depth estimation errors and by uneven ground surfaces that reduce measurement accuracy (Zhang et al., 2023). In the present study, this limitation was mitigated by defining a base plane at the upper surface of the cultivation tank rather than using the greenhouse floor as the reference surface. This approach enabled more precise height estimation by accounting for local variation in pot elevation and inclination. In addition, the use of PREPs allowed automated adjustment of the reference plane and facilitated extraction not only of plant height, but also of canopy coverage and volume index, thereby expanding the phenotypic descriptors available for plant-level analysis (Itoh et al., 2024).

The results further showed that HTP-derived crop traits were closely associated with biomass accumulation, supporting their utility as predictors in a phenotyping-based biomass modelling framework. Among the evaluated traits and trait combinations, models that incorporated structural information beyond canopy coverage alone generally showed stronger relationships with biomass. This tendency was particularly evident when volume index was included, suggesting that three-dimensional canopy characteristics contributed additional explanatory power. Because canopy coverage primarily represents horizontal canopy extent, it may not fully capture differences in vertical development or foliage density among plants. By contrast, volume index provides a proxy for three-dimensional canopy structure and may therefore better reflect total vegetative development. The stronger relationship observed for volume-based predictors suggests that biomass accumulation in buckwheat depends not only on horizontal canopy expansion, but also on the spatial arrangement of canopy tissues Because biomass estimation usually requires destructive measurement, volume index derived from the multi-camera system provides a non-destructive alternative that can be repeatedly measured throughout the growth period, thereby offering insight into the growth characteristics of buckwheat plants. Genotypic differences in canopy architecture appeared to influence both trait estimation and biomass relationships. “Kitamitsuki” tended to show greater plant height than “18sd-1”, which is consistent with its more vigorous canopy development observed during later growth stages.

The decline in biomass accumulation observed in the later growth stage of “18sd-1” was consistent with previous observations in some buckwheat varieties, where biomass decreases during the latter part of the generative period as reproductive development progresses (Potapov et al., 2017). Previous studies in Tartary buckwheat have reported that longer stems with more uniform elongation are associated with greater lodging susceptibility, whereas shorter or differently elongated stem types may show distinct structural behaviour (Hagiwara et al., 1999). In the present study, the taller and more laterally extended canopy of “Kitamitsuki” may explain both its greater biomass and the somewhat lower agreement between manual and image-derived height estimates, because the imaging system captures canopy extremities whereas manual measurements tend to emphasize the main stem. In addition, differences in stem thickness, leaf layering, and vertical growth strategy may have contributed to genotype-specific canopy architecture.

However, for buckwheat, the pre- and post-flowering stages for buckwheat did not produce a clear phenological difference in the correlation with HTP-derived traits and biomass. This is because buckwheat flowering prolongs during the maturity stage and by the time flowering occurs, the development in most parts of the crops has reached maturity stage. Thus, it is imperative to further subdivide the flowering into either early or mid-flowering using the specific flowering dates for each of the individual buckwheat and determine the effect of early and mid-flowering stages on the growth of buckwheat. By separating into early and mid-growth stages, it was shown that early flowering was characterised by slower growth while the mid-flowering stages, rapid and increased growth occurred leading to increased biomass and higher HTP-derived traits. were also observed.

The gradient between biomass and volume index was higher for “18sd-1” than for “Kitamitsuki”, whereas the gradient between biomass and canopy coverage was similar between the two genotypes. This suggests that canopy coverage captured similar horizontal expansion in both genotypes, while volume index reflected genotype-specific differences in vertical growth and canopy structure. Biologically, volume index can be interpreted as an image-derived indicator of overall three-dimensional canopy development. As plants grow, increases in plant height together with horizontal canopy expansion increase the reconstructed canopy space and consequently the volume index. In contrast, canopy coverage primarily represents the projected horizontal extent of vegetation and does not explicitly describe vertical canopy development. Volume index therefore provides complementary structural information related to plant stature and canopy development, which may explain its strong relationship with biomass observed in this study. Nevertheless, because it is derived from the reconstructed canopy surface rather than from direct measurement of the physical volume occupied by plant tissues, it should be regarded as a relative structural index rather than as actual canopy volume. Importantly, the difference in the biomass–volume index slopes indicate that the quantitative conversion of volume index to biomass may depend on genotype or canopy architecture. Therefore, the present results should not be interpreted as establishing a single genotype-independent calibration between volume index and biomass. Rather, they demonstrate the potential of volume index as a structural predictor of biomass, for which genotype or architecture-specific calibration may be required. Because only two contrasting genotypes were examined in this study, further evaluation across a broader range of buckwheat germplasm is necessary to determine whether a common calibration can be developed or whether genotype-specific models provide more robust biomass estimates. As a proxy for comparison to the currently utilised techniques, the integration of image-derived phenotypic traits with a simple radiation use efficiency (RUE) framework for biomass estimation was incorporated. The approach was based on the Monteith concept, whereby biomass accumulation is related to the amount of intercepted radiation and the efficiency with which that radiation is converted into plant biomass. In the present implementation, canopy coverage was used as a practical proxy for radiation interception. Although this simplification does not explicitly account for canopy depth or leaf area distribution, it provided a biologically interpretable basis for relating phenotyping data to biomass accumulation. The observed improvement in model performance when developmental stage was incorporated indicates that RUE in buckwheat is not constant throughout growth but instead varies with phenological progression.

Among the HTP-derived crop traits estimated from the phenotyping system, volume index showed a consistently high correlation with biomass in both genotypes, highlighting its value as a phenotypic predictor of plant growth. This strong relationship is likely because volume index captures the three-dimensional nature of canopy development by integrating both horizontal expansion and vertical growth. In contrast to canopy coverage, which primarily represents projected ground cover, volume index reflects a broader range of structural features, including canopy depth, plant stature, and the distribution of vegetative tissues within the canopy space. Such features are more closely aligned with actual biomass accumulation, since aboveground dry matter is determined by the total amount of plant material rather than by horizontal spread alone. The results therefore suggest that volume index is not merely a geometric descriptor, but a biologically relevant indicator of whole-plant development. The strong relationships observed in both “18sd-1” and “Kitamitsuki” support the potential of volume index as a non-destructive trait for biomass estimation, although the genotype-specific differences in regression slopes indicate that calibration across a broader range of canopy architectures is required before general application.

Partitioning the growth period into a 2-stage model consisting of pre- and post-flowering stages improved RUE estimation, and 3-stage model that involved further subdivision of the pre-flowering period into early and mid-stages provided an additional improvement in biomass estimation. The low apparent RUE observed during the early pre-flowering stage is likely attributable to incomplete canopy closure. At this stage, a substantial proportion of incoming radiation is intercepted by the soil rather than by the plant canopy, resulting in lower apparent efficiency of radiation conversion. As canopy development progresses, leaf area and canopy density increase, and the crop approaches a state of higher light interception and greater photosynthetic capacity. This likely explains the markedly higher RUE observed during the mid pre-flowering stage. The estimated RUE values in the mid and post-flowering stages were within the range previously reported for buckwheat, approximately 0.9–1.4 (Wan et al., 2023), supporting the physiological plausibility of the present approach.

The stage dependence of biomass accumulation observed here is also consistent with earlier physiological modelling studies of buckwheat. Kajfež-Bogataj and Hočevar, 1989 showed that dry matter production in buckwheat can be predicted from canopy photosynthesis as a function of shortwave radiation and temperature, with the contribution of plant organs varying across development. In particular, dry matter accumulation before flowering is dominated by vegetative organs such as stalks, whereas reproductive structures make a larger contribution after flowering. This developmental shift provides a physiological basis for the stage-specific RUE values observed in the present study. The decline in biomass accumulation observed in the later stage of “18sd-1” may likewise reflect changes in assimilate partitioning and senescence during the transition from flowering to maturity, as reported previously for some buckwheat varieties (Kajfež-Bogataj and Hočevar, 1989; Potapov et al., 2017).

From a breeding perspective, the ability to estimate biomass non-destructively at the individual plant level around the flowering stage is particularly valuable in buckwheat. Because buckwheat is an outcrossing species with heteromorphic self-incompatibility, breeding progress through conventional selection is inherently complex (Ohsawa, 2020). Although mass selection has historically been used to improve populations, individual plant selection remains important for identifying superior phenotypes and improving the efficiency of breeding programs (Yano et al., 2002). In Hokkaido, for example, the development of “Kitawasesoba” involved repeated selection from large populations over multiple generations (Honda and Abe, 1992). In this context, a high-throughput phenotyping system capable of quantifying plant height, canopy development, and biomass-related traits at the individual level could substantially improve selection efficiency. The present study therefore provides not only a modelling framework for biomass estimation, but also a practical phenotyping tool with potential utility in buckwheat breeding.

More broadly, the proposed approach occupies an intermediate position between highly detailed process-based crop models and purely empirical statistical models. Process-based models such as WOFOST describe crop growth through detailed representation of phenology, assimilation, respiration, partitioning, and transpiration processes (de Wit et al., 2019) whereas simpler statistical approaches often rely on agrometeorological or vegetation-index relationships without explicit physiological interpretation (Ji et al., 2022). The model presented here was intentionally simple, in line with the principle that biomass and yield prediction models should only increase in complexity when such additions improve predictive performance (Sinha and Mohanty, 2023). By combining image-derived phenotypes, the method retains biological interpretability while remaining practical for high-throughput application. This is conceptually similar to light use efficiency approaches that have been used in large-scale remote-sensing studies of crop biomass, including improved CASA-based modelling of regional wheat production (Huang et al., 2012).

It should be emphasized that the RUE estimated in this study was based on an image-derived approximation of radiation interception rather than on direct measurements of intercepted radiation. Canopy coverage represents the horizontally projected fraction of vegetation and was therefore used as a practical proxy for the fraction of incident radiation intercepted by the canopy. However, this approximation does not account explicitly for differences in leaf angle, leaf area distribution, canopy depth, or self-shading, which may vary between genotypes and developmental stages. In addition, greenhouse transmissivity was represented by a fixed value of approximately 90%, although actual transmission can vary temporally with solar angle and greenhouse conditions. Consequently, the resulting RUE values should be interpreted as apparent RUE estimates within the simplified modelling framework used here rather than as direct physiological measurements of RUE. Direct measurements of transmitted and intercepted radiation within the canopy would be valuable in future studies to validate and refine this approach.

Despite these advantages, several limitations should be acknowledged. First, the present study was conducted under greenhouse conditions, where environmental variability and canopy-background complexity were lower than would be expected under open-field conditions. Furthermore, reconstruction quality represents an important limitation of the proposed phenotyping workflow. During image acquisition, strong wind occasionally passed through the greenhouse and caused movement of the buckwheat plants. Because SfM/MVS reconstruction assumes that the imaged objects remain sufficiently stable among overlapping images, such movement can cause image blur and inconsistencies among images, resulting in incomplete three-dimensional reconstruction. This problem was most pronounced at 24 DAP, for which reconstruction quality was insufficient and the complete dataset was excluded from further analysis. Reconstruction failures of this magnitude were otherwise infrequent. However, at some other measurement dates, individual plants showed localized reconstruction errors that resulted in temporary non-biological decreases in estimated volume index, as illustrated in **Figure 9**. These observations indicate that volume index is more sensitive to reconstruction errors than canopy coverage because it depends on the accuracy of the reconstructed three-dimensional canopy surface. Although this problem occurred only occasionally under the greenhouse conditions used in this study, plant movement caused by wind is expected to represent a greater challenge under outdoor field conditions. Future application of the system should therefore consider image acquisition under low-wind conditions and additional quality-control procedures for identifying unreliable three-dimensional reconstructions. Thirdly, only two genotypes with contrasting plant architectures were evaluated in the present study. The observed difference in the slopes of the biomass–volume index relationships suggest that canopy architecture may influence the conversion of image-derived structural traits to biomass. Consequently, the generality of a common volume index–biomass calibration across buckwheat genotypes remains to be established. In addition, the stage partitioning strategy was derived from the observed developmental patterns of these two genotypes and may require further validation across a broader range of germplasm and environments. Future work should therefore evaluate the transferability of this framework to field-grown buckwheat, test genotype-specific and common calibration approaches using more diverse germplasm and assess whether additional phenotypic descriptors can further improve biomass prediction. Final limitation is that the relationships developed in this study were evaluated using the same dataset from which they were fitted, and neither cross-validation nor independent validation was performed. Consequently, the reported R^2^ and RMSE values should be interpreted as measures of model fit rather than predictive performance on unseen data. Future studies should validate these relationships using independent datasets collected from additional genotypes, growing seasons, and environmental conditions to determine their robustness and generalizability for biomass prediction.

**Figure 9:**
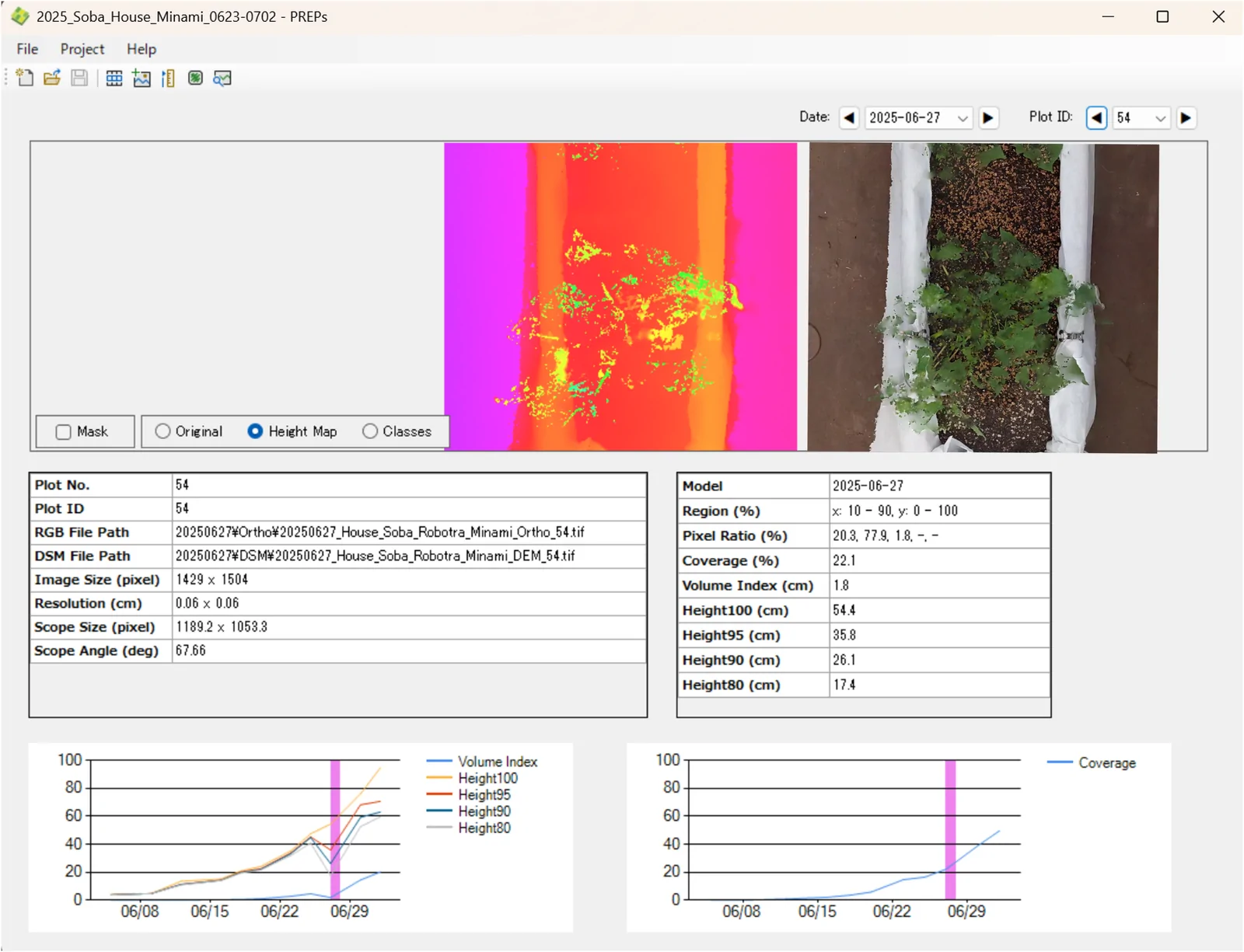
Example of PREPs-processed images affected by failed 3D reconstruction, leading to inaccurate volume index estimation. The graph shows a sharp decrease in volume index, whereas canopy coverage was less affected.

Thirdly, the stage partitioning strategy was derived from the observed development pattern of the two genotypes examined here and may require further validation across a broader range of germplasm and environments. Future work should therefore evaluate the transferability of this framework to field-grown buckwheat, test its robustness across more diverse genotypes, and assess whether additional phenotypic descriptors can further improve biomass prediction.

Overall, the present findings indicate that high-throughput image-based phenotyping of individual of can be used to predict biomass at the individual-plant level in buckwheat. This integration of phenotyping and growth modelling provides a practical basis for repeated, non-destructive assessment of biomass-related traits and may contribute to more efficient selection in buckwheat breeding programs.

## 5 Conclusion

In this study, we developed a non-destructive approach for estimating individual-plant biomass in buckwheat using HTP-derived crop traits obtained from a tractor-mounted multi-camera phenotyping system. The system enabled extraction of plant height, canopy coverage, and volume index at the individual-plant level. Image-derived plant height showed strong agreement with manual measurements, with coefficients of determination of 0.95 and 0.93 for “18sd-1” and “Kitamitsuki”, respectively, confirming the reliability of the phenotyping platform for repeated monitoring of buckwheat growth.

HTP-derived crop traits were strongly associated with measured biomass and provided a practical basis for biomass estimation. Volume index showed a similar accuracy as that of the conventional coverage-based RUE framework, indicating that three-dimensional canopy structure is an important descriptor of biomass accumulation in buckwheat. Incorporating developmental stage further improved model performance. The three-stage model, which divided growth into early pre-flowering, mid pre-flowering, and post-flowering stages, provided better biomass estimation than the one-stage and two-stage models.

Overall, the present findings indicate that high-throughput image-based phenotyping can be used to predict biomass at the individual-plant level in buckwheat. This integration of phenotyping and growth modelling provides a practical basis for repeated, non-destructive assessment of biomass-related traits and may contribute to more efficient selection in buckwheat breeding programs.

Future studies should evaluate the applicability of this approach under field conditions and across a broader range of genotypes to further assess its robustness for buckwheat phenotyping and breeding.

## Data Availability

The original contributions presented in the study are included in the article/**Supplementary Material**. Further inquiries can be directed to the corresponding author.

## References

[B1] Amelchanka S. L. Kreuzer M. Leiber F. (2010). Utility of buckwheat (*Fagopyrum esculentum* Moench) as feed: Effects of forage and grain on *in vitro* ruminal fermentation and performance of dairy cows. Anim. Feed Sci. Technol. 155, 111–121. doi: 10.1016/j.anifeedsci.2009.10.00742574925

[B2] Antala M. Kovar M. Sporinová L. Filacek A. Juszczak R. Zivcak M. . (2025). High-throughput phenotyping of buckwheat (Fagopyrum esculentum Moench.) genotypes under water stress: Exploring drought resistance for sustainable agriculture. BMC Plant Biol. 25, 444. doi: 10.1186/s12870-025-06429-6 40200140 PMC11978128

[B3] Arduini I. Masoni A. Mariotti M. (2016). A growth scale for the phasic development of common buckwheat. Acta Agriculturae Scandinavica Section B. — Soil Plant Sci. 66, 215–228. doi: 10.1080/09064710.2015.108758737339054

[B4] Bergjord Olsen A. K. Persson T. de Wit A. Nkurunziza L. Sindhøj E. Eckersten H. (2018). Estimating winter survival of winter wheat by simulations of plant frost tolerance. J. Agron. Crop Sci. 204, 62–73. doi: 10.1111/jac.1223840046247

[B5] Briak H. Kebede F. (2021). Wheat (Triticum aestivum) adaptability evaluation in a semi-arid region of Central Morocco using APSIM model. Sci. Rep. 11, 23173. doi: 10.1038/s41598-021-02668-3 34848819 PMC8632900

[B6] Brown H. Huth N. Holzworth D. (2018). Crop model improvement in APSIM: Using wheat as a case study. Eur. J. Agronomy Recent Adv. Crop Model. to support Sustain. Agric. production Food. Secur. under Global Change 100, 141–150. doi: 10.1016/j.eja.2018.02.00242574925

[B7] Chen J. Zhang P. Liu J. Deng J. Su W. Wang P. . (2024). Study on the impact of low-temperature stress on winter wheat based on multi-model coupling. Food Energy Secur. 13, e543. doi: 10.1002/fes3.54342587375

[B8] Dewenam L. E. F. Er-Raki S. Ezzahar J. Chehbouni A. (2021). Performance evaluation of the WOFOST model for estimating evapotranspiration, soil water content, grain yield and total above-ground biomass of winter wheat in Tensift Al Haouz (Morocco): Application to yield gap estimation. Agronomy 11, 2480. doi: 10.3390/agronomy1112248030654563

[B9] de Wit A. Boogaard H. Fumagalli D. Janssen S. Knapen R. van Kraalingen D. . (2019). 25 years of the WOFOST cropping systems model. Agric. Syst. 168, 154–167. doi: 10.1016/j.agsy.2018.06.01842574925

[B10] Filho A. C. Neu I. M. M. Souza J. M. D. Pezzini R. V. Dumke G. E. Somavilla F. M. (2020). Plot size, number of treatments and replicates and experimental precision in buckwheat. Rev. Caatinga 33, 1131–1139. doi: 10.1590/1983-21252020v33n428rc

[B11] Hagiwara M. Izusawa H. Inoue N. Matano T. (1999). Varietal differences of shoot growth characters related to lodging in Tartary buckwheat 16, 67–72.

[B12] Honda Y. Abe N. (1992). “ Breeding of new buckwheat cultivars in Hokkaido, Japan”, in: Proc. Of 5th Int. Sympo. on buckwheat (Taiyuan: Citeseer).

[B13] Huang C. Asner G. P. Barger N. N. (2012). Modeling regional variation in net primary production of pinyon–juniper ecosystems. Ecol. Modell. 227, 82–92. doi: 10.1016/j.ecolmodel.2011.12.01642574925

[B14] Hung P. V. Maeda T. Morita N. (2009). Buckwheat starch: Structure and characteristics – A review.3(1), 23–28.

[B15] Itoh A. Njane S. N. Hirafuji M. Guo W. (2024). PREPs: An open-source software for high-throughput field plant phenotyping. Plant Phenomics 6, 221. doi: 10.34133/plantphenomics.0221 39130162 PMC11310773

[B16] Ji F. Meng J. Cheng Z. Fang H. Wang Y. (2022). Crop yield estimation at field scales by assimilating time series of Sentinel-2 data into a modified CASA-WOFOST coupled model. IEEE Trans. Geosci. Remote Sens. 60, 1–14. doi: 10.1109/TGRS.2020.304710225079929

[B17] Jung G. H. Kim S. L. Kim M. J. Kim S. K. Park J. H. Kim C. G. . (2015). Effect of sowing time on buckwheat (Fagopyrum esculentum Moench) growth and yield in central Korea. J. Crop Sci. Biotechnol. 18, 285–293. doi: 10.1007/s12892-015-0117-630311153

[B18] Kajfež-Bogataj L. Hočevar A. (1989). Modelling of net photosynthetic productivity for buckwheat (*Fagopyrum esculentum*) moench. Agric. For. Meteorol. 44, 233–244. doi: 10.1016/0168-1923(89)90019-1

[B19] Landsberg J. J. Hingston F. J. (1996). Evaluating a simple radiation/dry matter conversion model using data from Eucalyptus globulus plantations in Western Australia. Tree Physiol. 16, 801–808. doi: 10.1093/treephys/16.10.801 14871669

[B20] Li Z. Nie Z. Li G. Li Z. Nie Z. Li G. (2024). Integrating crop modeling and machine learning for the improved prediction of dryland wheat yield. Agronomy 14, 777. doi: 10.3390/agronomy1404077730654563

[B21] Liu Y. Hatou K. Aihara T. Kurose S. Akiyama T. Kohno Y. . (2021). A robust vegetation index based on different UAV RGB images to estimate SPAD values of naked barley leaves. Remote Sens. 13, 686. doi: 10.3390/rs1304068630654563

[B22] Mikami T. Motonishi S. Tsutsui S. (2018). Production, uses and cultivars of common buckwheat in Japan: An overview. Acta agriculturae Slovenica 111, 511–517. doi: 10.14720/aas.2018.111.2.23

[B23] Monteith J. L. (1972). Solar radiation and productivity in tropical ecosystems. J. Appl. Ecol. 9, 747–766. doi: 10.2307/2401901

[B24] Njane S. N. Itoh A. Yoshida M. Shuri T. Tsuchiya S. Tsuji H. (2024). A novel tractor-mounted multi-camera system for precise high-throughput phenotyping. Journal of the Japanese Society of Agricultural Machinery and Food Engineers 86(3), 172–187. doi: 10.11357/jsamfe.86.3_172

[B25] Ohsawa R. (2020). Current status and prospects of common buckwheat breeding in Japan. Breed. Sci. 70, 3–12. doi: 10.1270/jsbbs.19108 32351299 PMC7180140

[B26] Osman A. M. (1971). Dry-matter production of a wheat crop in relation to light interception and photosynthetic capacity of leaves. Ann. Bot. 35, 1017–1035. doi: 10.1093/oxfordjournals.aob.a08453840388063

[B27] Potapov K. O. Kadyrova L. R. Galiullina G. N. Kadyrova F. Z. Khusnutdinova A. T. Nikiforova I. Y. (2017). Morphological features and economic value of buckwheat varieties with physiological determination of growth. J. Pharm. Res. 11, 1252–1256.

[B28] Sinha N. K. Mohanty M. (2023). Basics of crop growth simulation modelling. In Agricultural Systems Modeling: Approaches in Vogue. (India: ICAR – Indian Institute of Rice Research, Hyderabad) pp. 183–201.

[B29] Suzuki T. Noda T. Morishita T. Ishiguro K. Otsuka S. Brunori A. (2020). Present status and future perspectives of breeding for buckwheat quality. Breed. Sci. 70, 48–66. doi: 10.1270/jsbbs.19018 32351304 PMC7180147

[B30] Suzuki T. Tsujimoto K. Maruo G. Hara T. Katsu K. Matsuura A. . (2023). Development of semidwarf breeding line with shattering resistance and preharvest-sprouting resistant in buckwheat (). Plant Breed. 142, 527–536. doi: 10.1111/pbr.1310140046247

[B31] Vieira M. I. de Melo-Abreu J. P. Ferreira M. E. Monteiro A. A. (2009). Dry matter and area partitioning, radiation interception and radiation-use efficiency in open-field bell pepper. Sci. Hortic. 121, 404–409. doi: 10.1016/j.scienta.2009.03.00742574925

[B32] Wan C. Gao S. Wang J. Lei X. Ge J. Tao J. . (2023). Optimal planting density combined with phosphorus input promotes common buckwheat resource use efficiency and productivity to increase grain yield. Agric. Water Manage. 287, 108468. doi: 10.1016/j.agwat.2023.10846842574925

[B33] Wu J. Zheng D. Wu Z. Song H. Zhang X. (2022). Prediction of buckwheat maturity in UAV-RGB images based on recursive feature elimination cross-validation: A case study in Jinzhong, Northern China. Plants 11, 3257. doi: 10.3390/plants11233257 36501299 PMC9737888

[B34] Yabe S. Hara T. Ueno M. Enoki H. Kimura T. Nishimura S. . (2018). Potential of genomic selection in mass selection breeding of an allogamous crop: An empirical study to increase yield of common buckwheat. Front. Plant Sci. 9. doi: 10.3389/fpls.2018.00276 29619035 PMC5871932

[B35] Yadav B. Krishnan P. Parihar C. M. Banerjee K. (2025). Modelling crop growth and soil hydrothermal regimes under conservation agriculture using APSIM-wheat. Sci. Rep. 15, 36362. doi: 10.1038/s41598-025-20211-6 41107323 PMC12534378

[B36] Yano K. Ohsawa R. Yonezawa K. (2002). Cost efficiency of spatial error control in single plant selection. Breed. Sci. 52, 177–184. doi: 10.1270/jsbbs.52.177

[B37] Zhang J. Xing W. Song X. Cui Y. Li W. Zheng D. . (2023). Buckwheat plant height estimation based on stereo vision and a regression convolutional neural network under field conditions. Agronomy 13, 2312. doi: 10.3390/agronomy1309231230654563

[B38] Zhang J. Yang G. Gao M. Zhang L. Chen W. Liu M. . (2025). Wheat yield prediction using an enhanced WOFOST with soil stratified hydrothermal module driven by GLASS and ERA5-land products over Yellow River Basin. Comput. Electron. Agric. 237, 110669. doi: 10.1016/j.compag.2025.11066942574925

