# Supplementary material for: Non-destructive estimation of individual buckwheat plant biomass using high-throughput phenotyping-derived canopy traits
Source: Front Plant Sci. 2026 Oct 8;17:1953140. doi: 10.3389/fpls.2026.1953140 (PMC13687041; doi:10.3389/fpls.2026.1953140)
Supplement: Supplementary file 1 [file DataSheet1.pdf]

Non-destructive estimation of individual buckwheat plant biomass using high-throughput phenotyping-derived canopy traits

Supplementary information

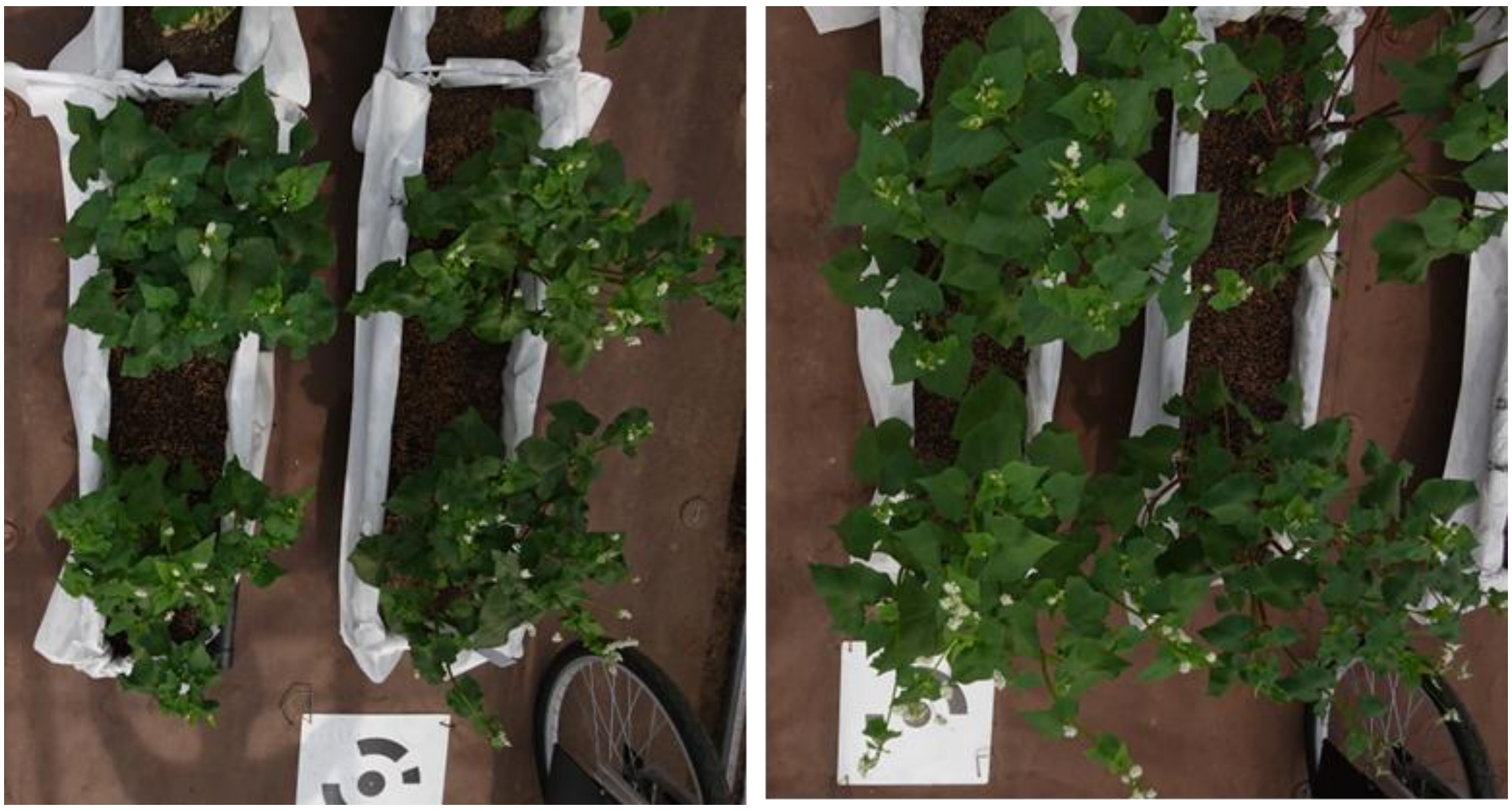

(a) (b)

Fig.1. Images of (a) “18sd-1” (b) “Kitamitsuki” taken at nadir by the multi-camera system.

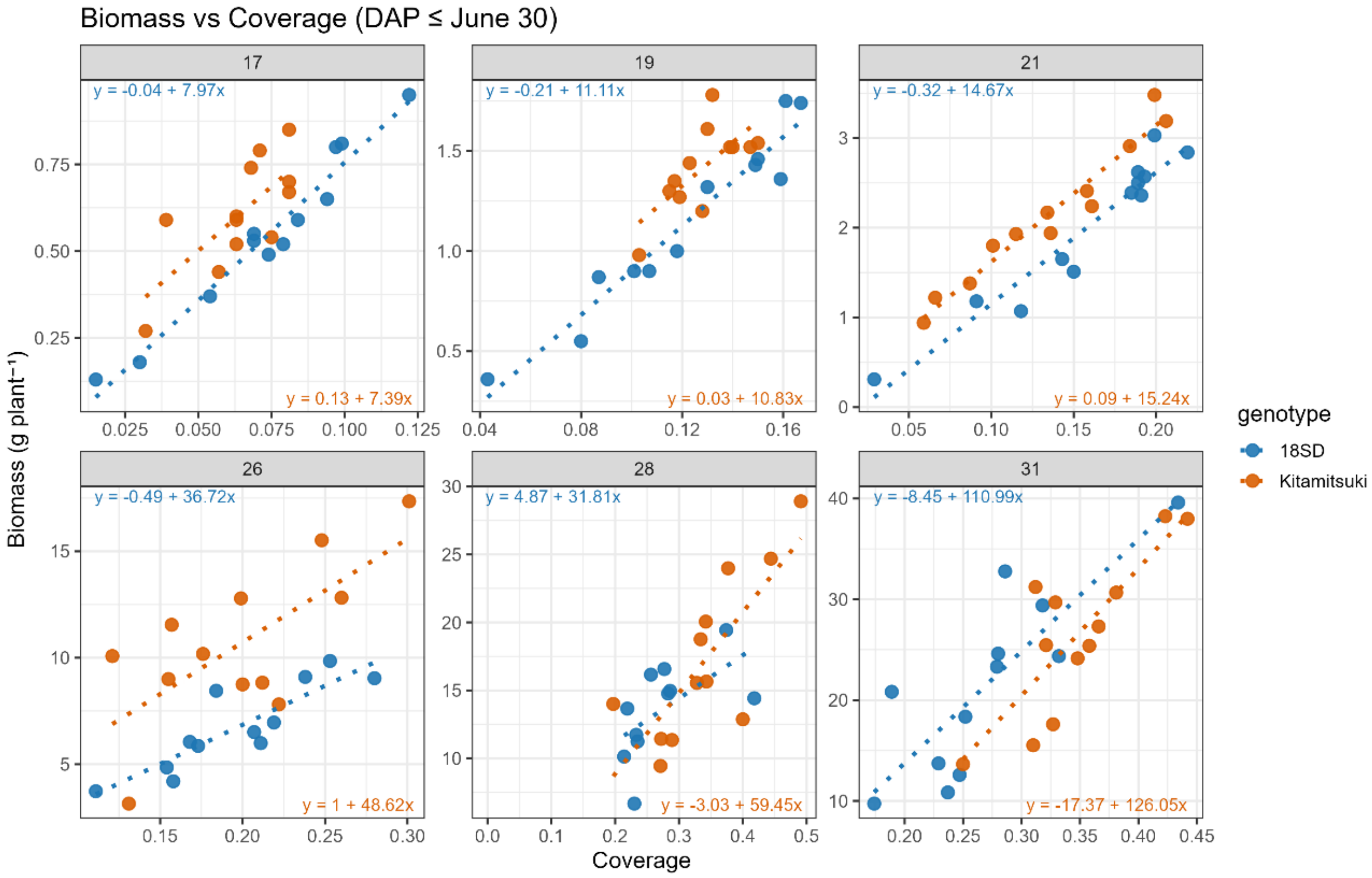

Fig. 2. Biomass correlation with Coverage with several days after planting

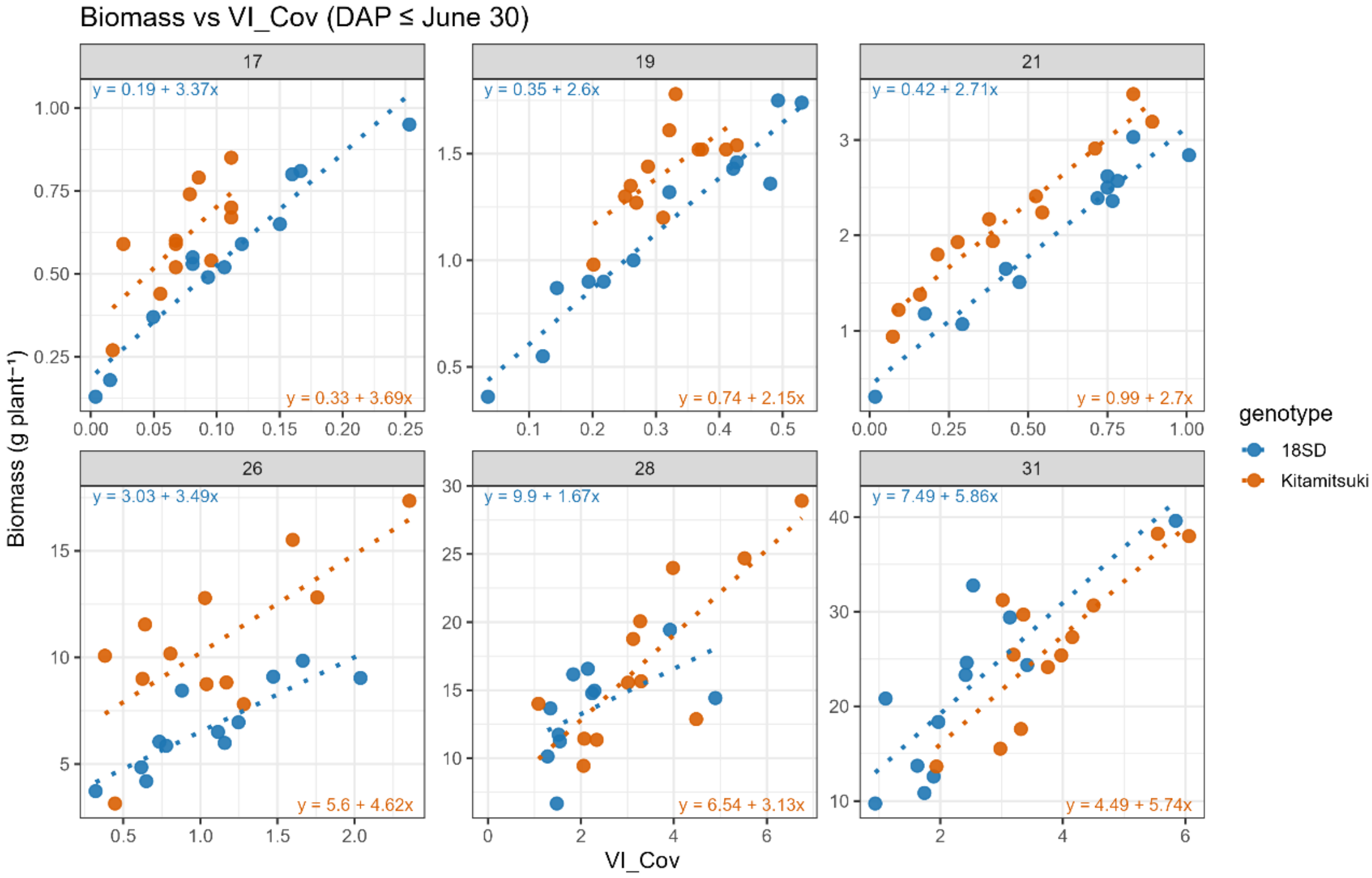

Fig. 3. Biomass correlation with Volume Index and Coverage

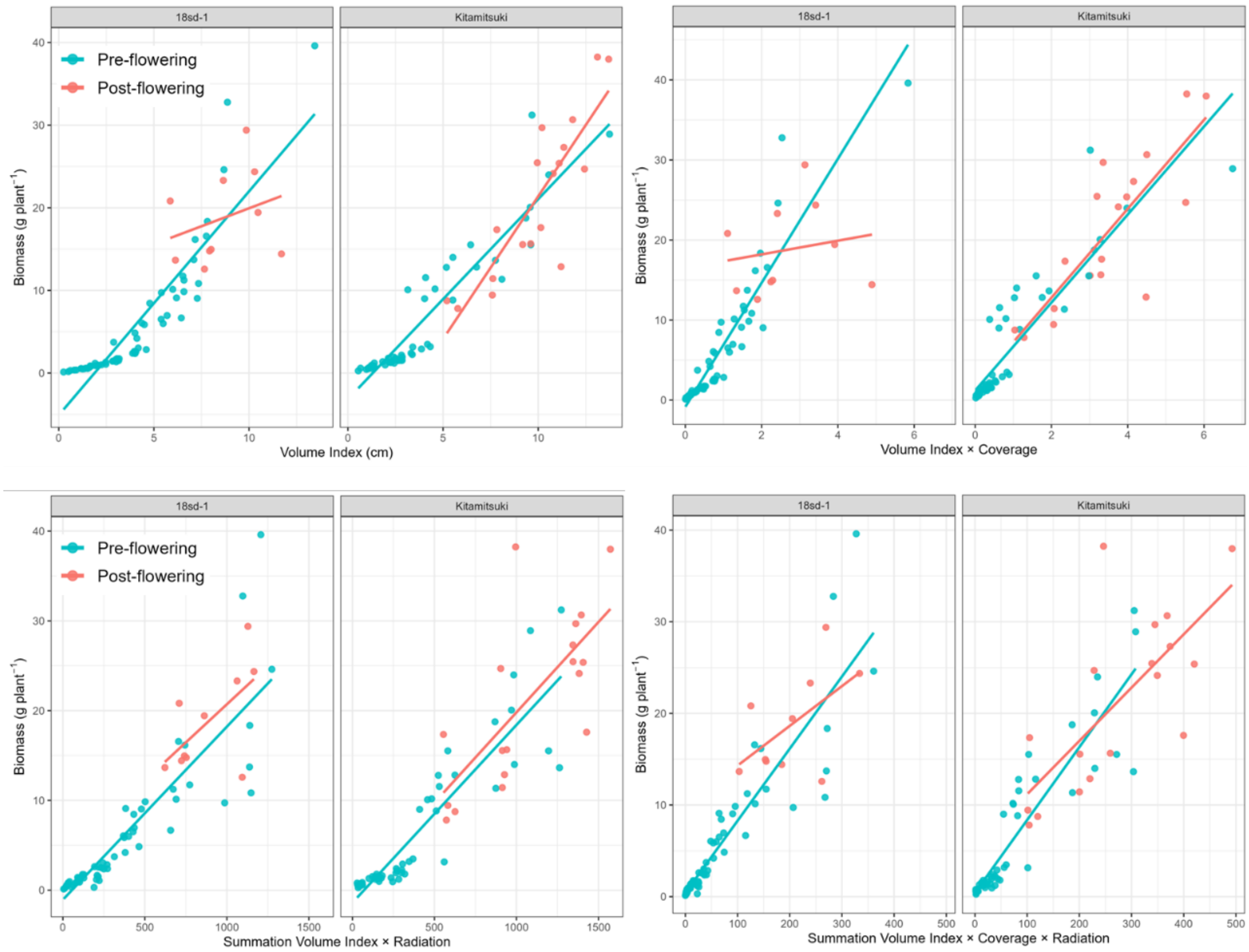

Fig. 4. Correlation between biomass with estimated crop traits and their combination with estimated radiation in pre and post flowering stages.

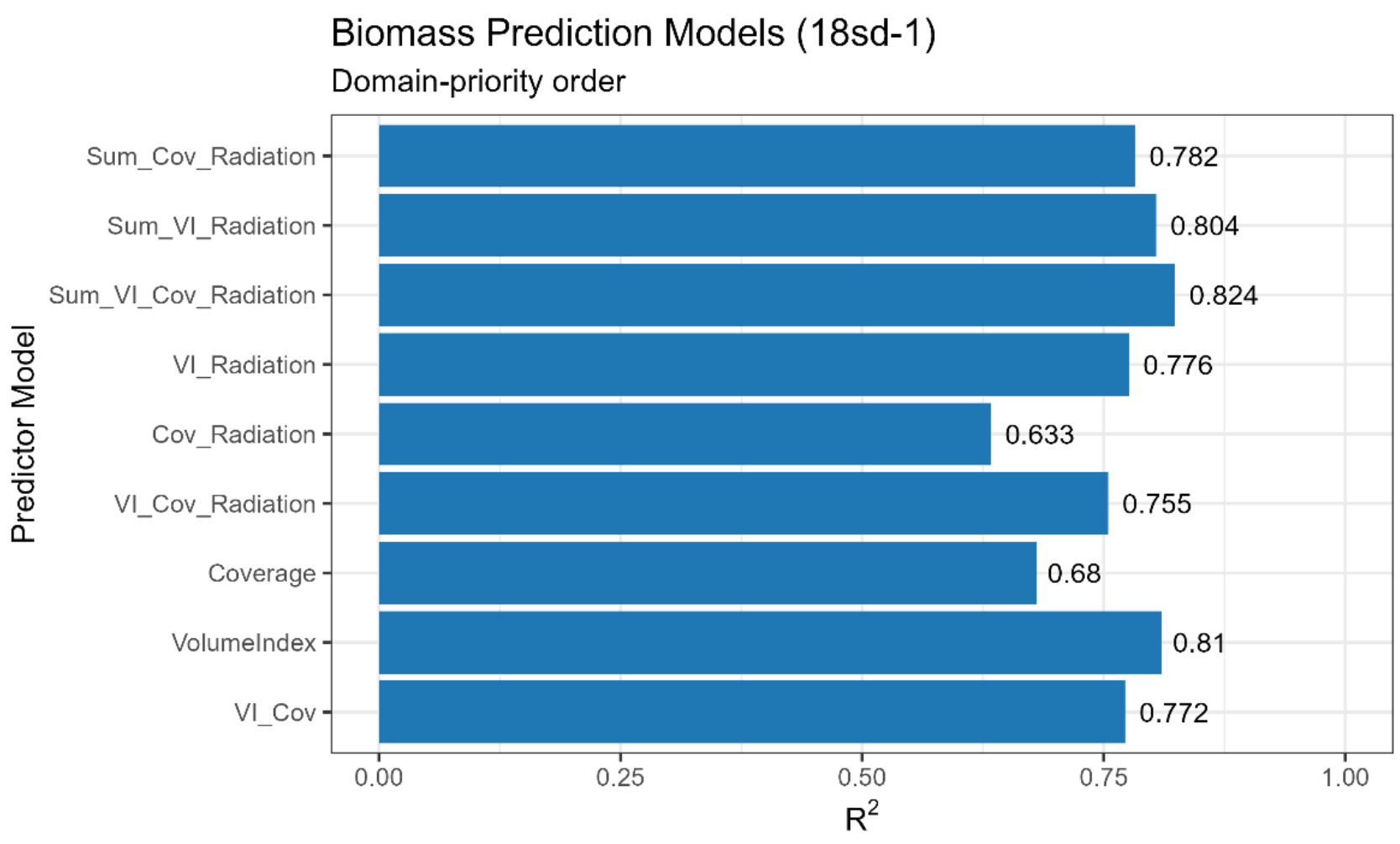

(a)

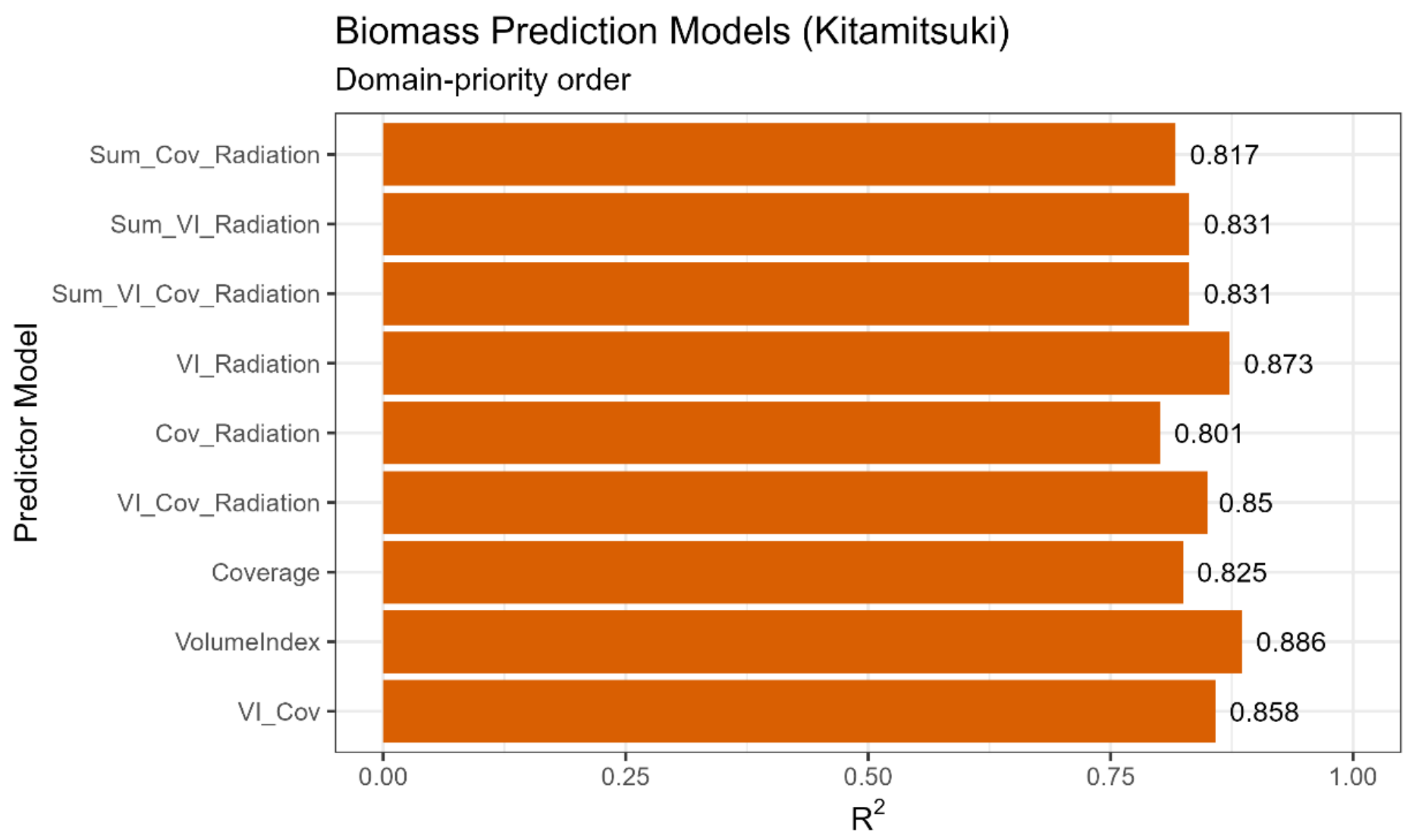

(b)

Fig. 5. Model ranking based on importance for RUE estimation derived from correlation between biomass and sensing derived parameters for (a) “18sd-1” and (b) “Kitamitsuki”.

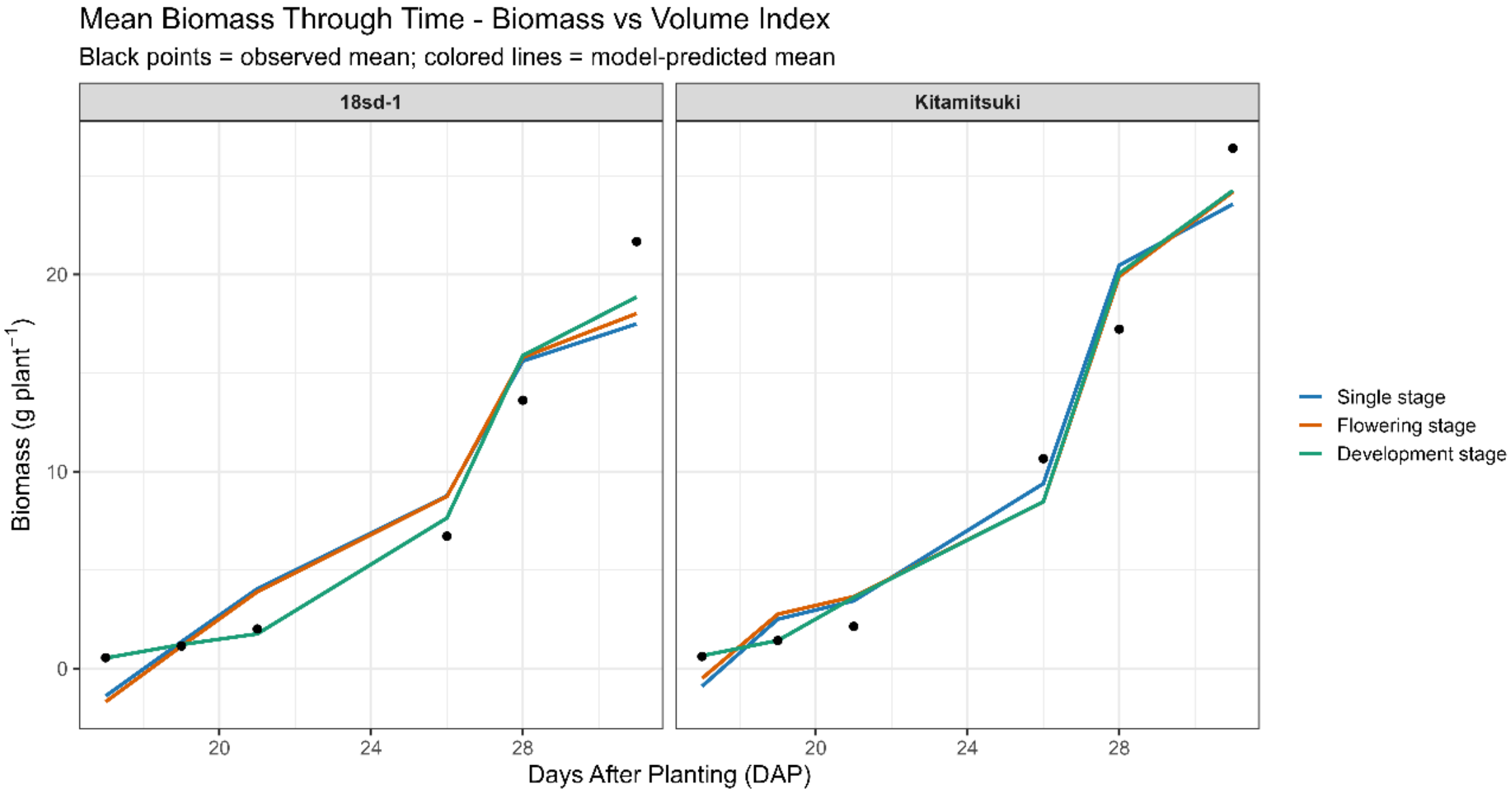

Fig. 6. Biomass prediction using Volume Index with close prediction observed especially in Kitamitsuki using the Development Stage

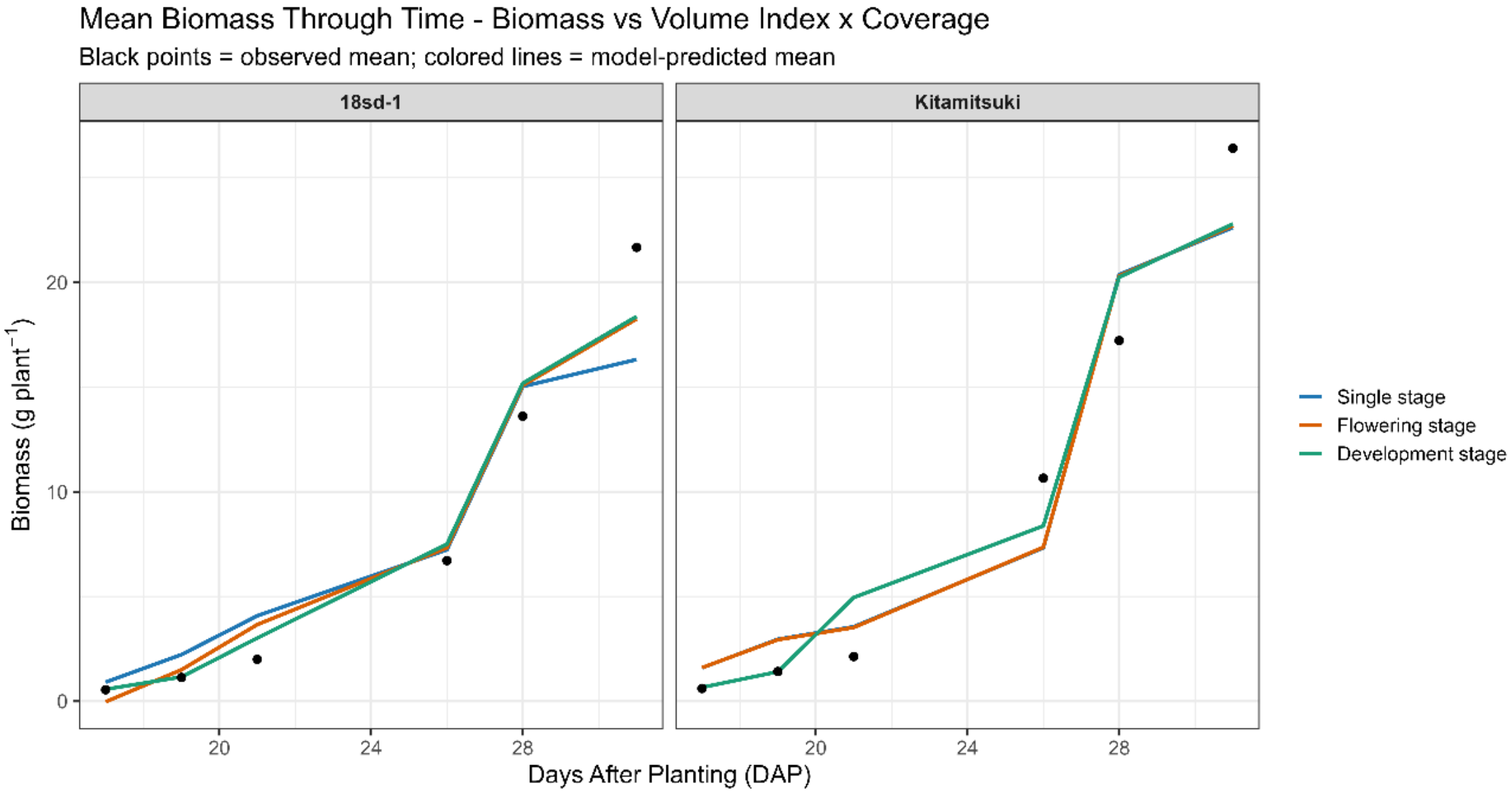

Fig.7. Biomass prediction using Volume Index × Coverage with close prediction observed especially in both varieties in all the stages.

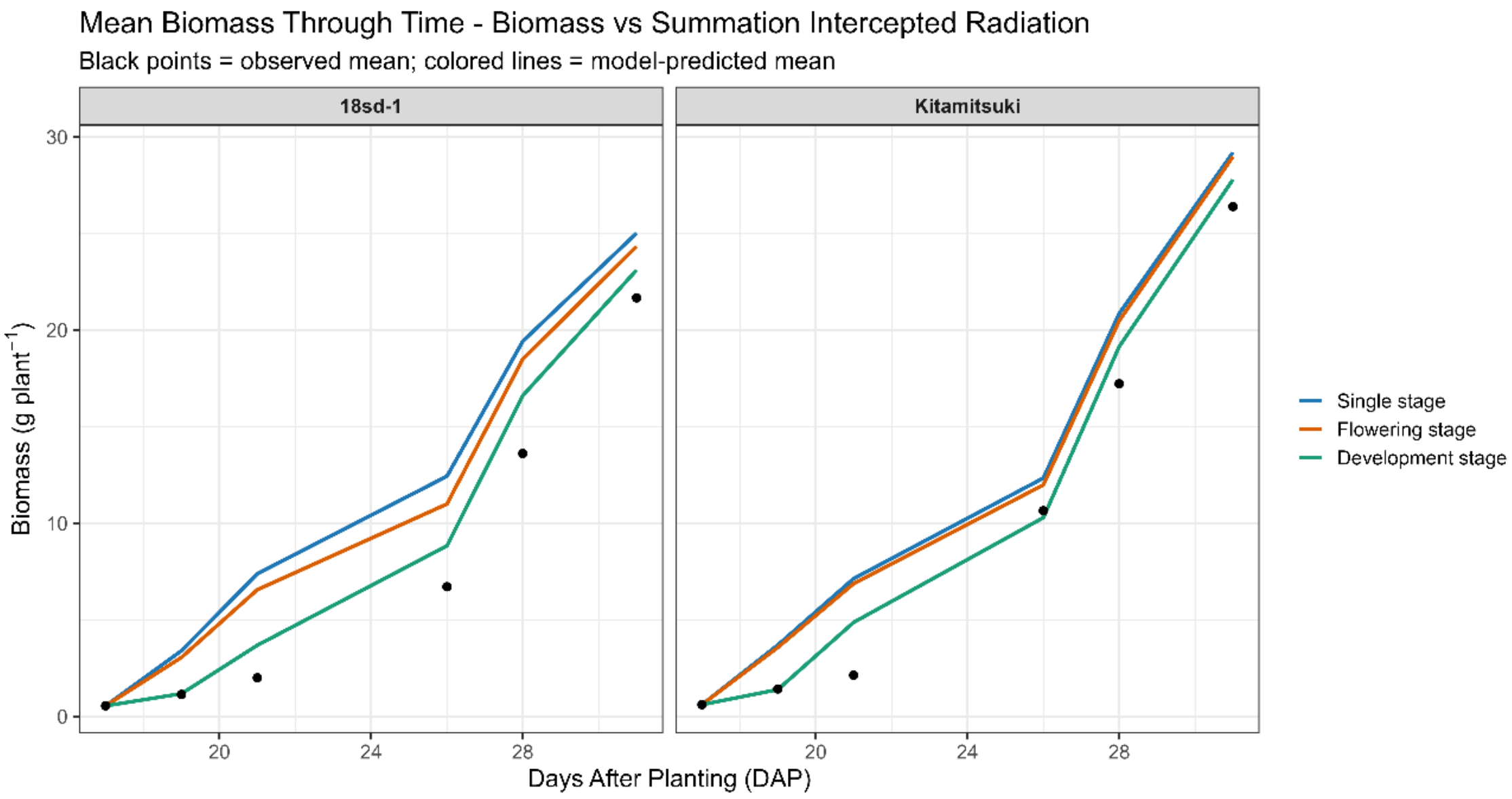

Fig. 8. Biomass prediction from the summation of the intercepted radiation with closes prediction observed using the development stage in both varieties.

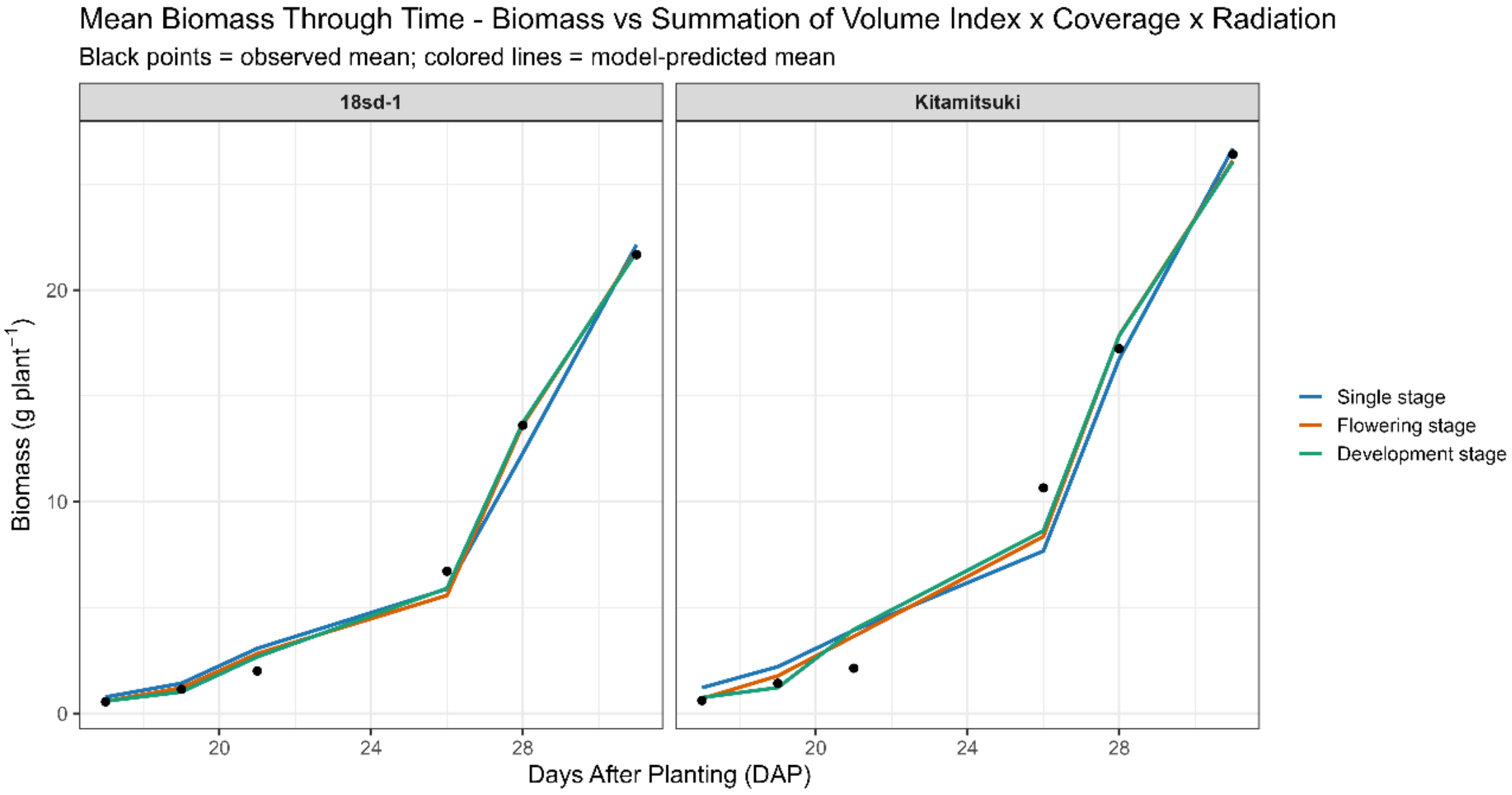

Fig. 9. Biomass prediction from the summation of Volume index × Coverage × radiation with closes prediction observed using the development stage in both varieties.

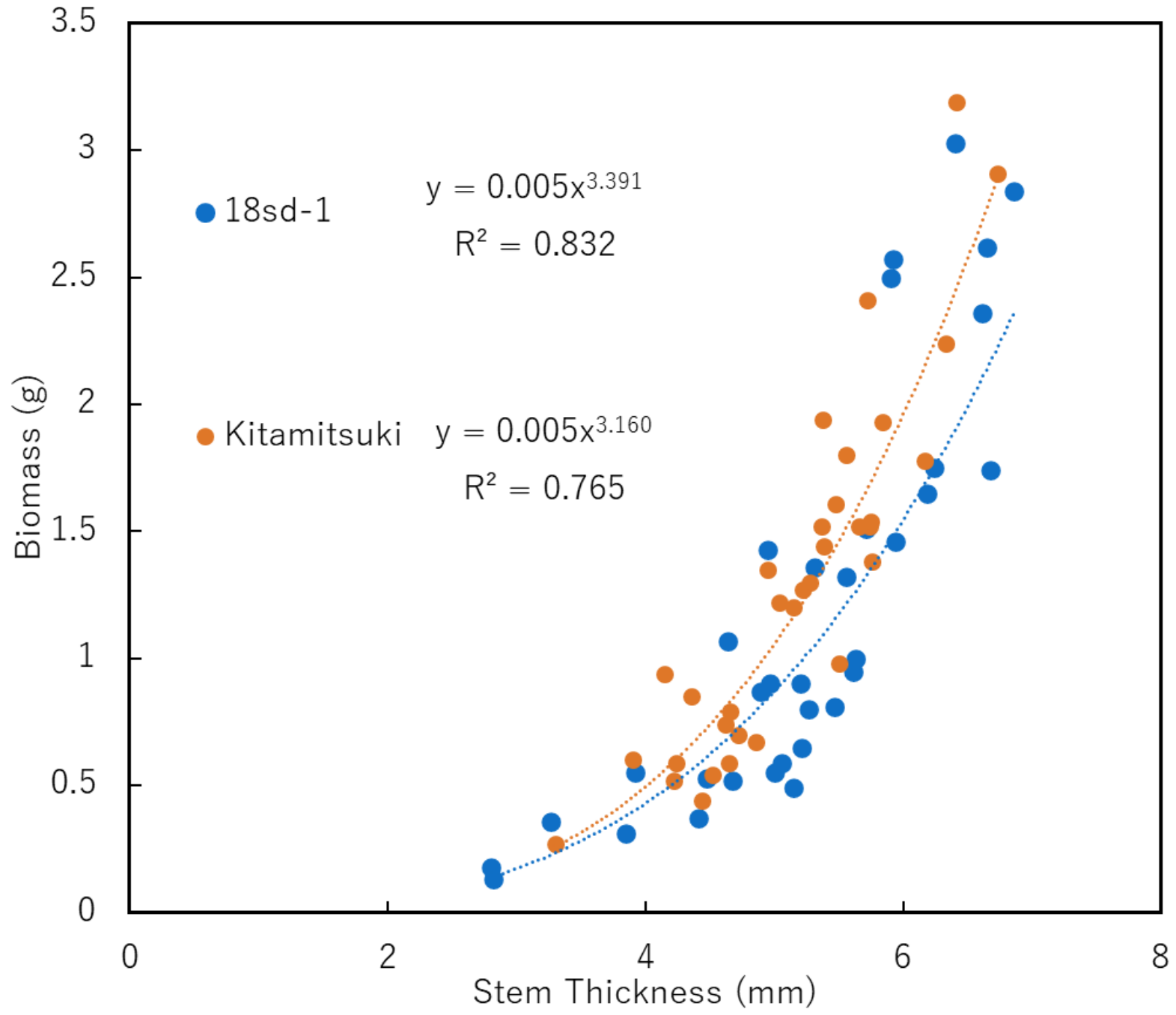

Fig. 10. Comparison of the correlation between Biomass and Stem thickness for both "18sd-1" and "Kitamitsuki".
